# Artificial intelligence-enabled monitoring of pesticide contamination in agricultural water systems: a comprehensive review of intelligent sensing technologies for water quality surveillance and environmental risk assessment

**DOI:** 10.1039/d6ra07513d

**Published:** 2026-11-03

**Authors:** Hayat Khan, Sani Isnain, Muhammad Anas, Abdullah H. Alshahri, Najam U. Saqib, R. Nawaz, Javed Iqbal, Waseem Ahmed Khattak, Khizar Hayat Khan

**Affiliations:** a Department of Chemical Engineering, College of Engineering, King Faisal University Al Ahsa 31982 Saudi Arabia khan@kfu.edu.sa; b Department of Software Engineering, Konya Technical University Konya 42250 Türkiye saniisnain50@gmail.com; c Department of Soil Science & Plant Nutrition, Selçuk University Konya 42079 Türkiye muhammed.anas@selcuk.edu.tr; d Department of Civil Engineering, College of Engineering, Taif University P.O. Box 11099 Taif City 21974 Saudi Arabia aalshahri@tu.edu.sa; e Department of Mechanical Engineering, Universiti Malaysia Pahang Al-Sultan Abdullah (UMPSA) 26600 Malaysia Najmusaqib77@gmail.com; f Center for Applied Mathematics and Bioinformatics (CAMB), Gulf University for Science and Technology 32093 Hawally Kuwait nawaz.r@gust.edu.kw; g Department of Botany, Bacha Khan University Charsadda Khyber Pakhtunkhwa 24420 Pakistan javed89qau@gmail.com; h Key Laboratory of Fruit Breeding Technology of the Ministry of Agriculture, Zhengzhou Fruit Research Institute, Chinese Academy of Agricultural Sciences Zhengzhou 450009 China waseemkhattack@bs.qau.edu.pk; i Department of Chemistry, Quaid-I-Azam University Islamabad Islamabad 45320 Pakistan khayat@chem.qau.edu.pk

## Abstract

Pesticide contamination in agricultural water systems threatens ecosystem health, water security, and sustainable agriculture. Conventional laboratory analyses are highly accurate but remain expensive, labor-intensive, and unsuitable for continuous field monitoring. This review summarizes recent advances in artificial intelligence (AI)-enabled sensing technologies for the real-time detection of pesticides in irrigation water, rivers, reservoirs, drainage canals, and groundwater. It covers electrochemical sensors, biosensors, hyperspectral imaging, fluorescence spectroscopy, optical sensing, and microfluidic platforms integrated with Internet of Things (IoT) networks, edge computing, and cloud analytics. Particular emphasis is placed on machine learning, deep learning, explainable AI, federated learning, graph neural networks, and multimodal data fusion for contaminant detection, prediction, and risk assessment. The distinctive contribution of this review is providing a pesticide-specific source-to-decision framework that links hydrological transport and chemical transformation to matrix-conditioned sensor responses, AI-assisted signal interpretation, validation maturity, and field deployment, thereby identifying where intelligent analytics can provide a meaningful advantage for pesticide surveillance in agricultural water systems.

## Abstract 2

Pesticide contamination in agricultural water systems threatens ecosystem health, water security, and sustainable agriculture. Image created in BioRender. Rashid, F. (2026) https://BioRender.com/cqp4kzs.

## Graphical abstract

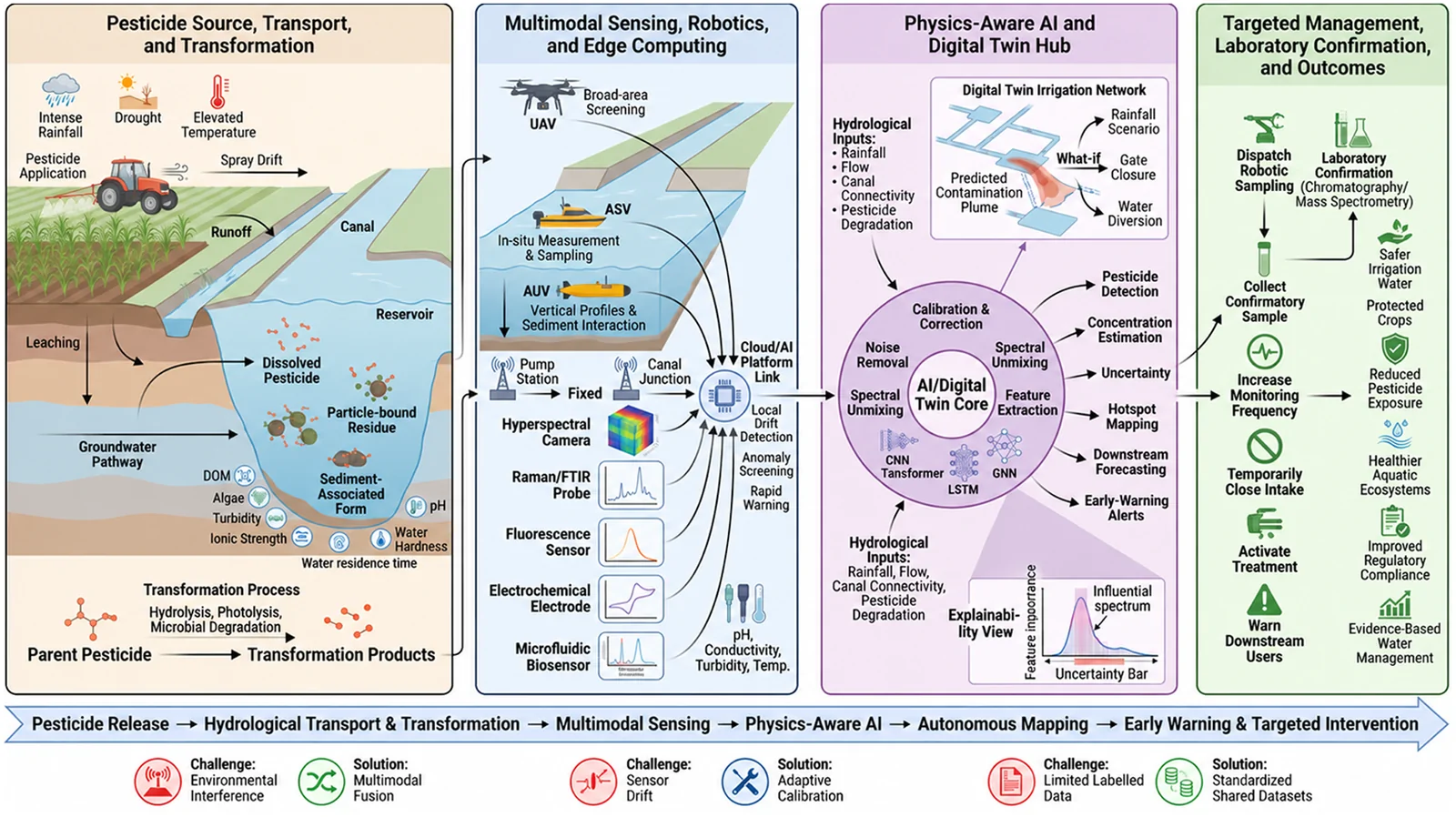

## 1 Introduction

The safety of irrigation water has emerged as a top-priority subject in agriculture in the twenty-first century. The use of agrochemicals has drastically increased as the world works hard to produce food to support ever-growing populations, hence the constant contamination of freshwater systems. Runoff and drainage water that contain a broad range of pesticides often enter irrigation canals, surface reservoirs, groundwater recharge basins and farm ponds. Such contaminants are dangerous to the health of crops, the biodiversity of the soil and, eventually, to human consumers.^1^ Pesticides get into irrigation systems *via* a complicated combination of both natural and artificial routes. The most evident path is surface runoff: pesticide residues from treated fields are washed directly into catchment basins and canals. Permeable soil can also permit the leaching of pesticides, which contaminate groundwater that is subsequently pumped to irrigate the land. Application causes spray drift, which allows aerosols and droplets to settle in open channels and reservoirs.^2^ Root zones dissolve pesticides, which are carried to ditches that supply irrigation networks by subsurface drainage (Fig. 1). Contaminants are also released directly through other sources, such as backflow events, the poor disposal of tank mixtures and poor irrigation infrastructure.^3^ These routes ensure a continuous flow of dissolved, colloidal and particulate forms of pesticides into water bodies, which are thus hard to detect and control. The knowledge of such entry points is critical in the context of developing specific monitoring systems and forecasting the dynamics of contamination.^4^

**Fig. 1:**
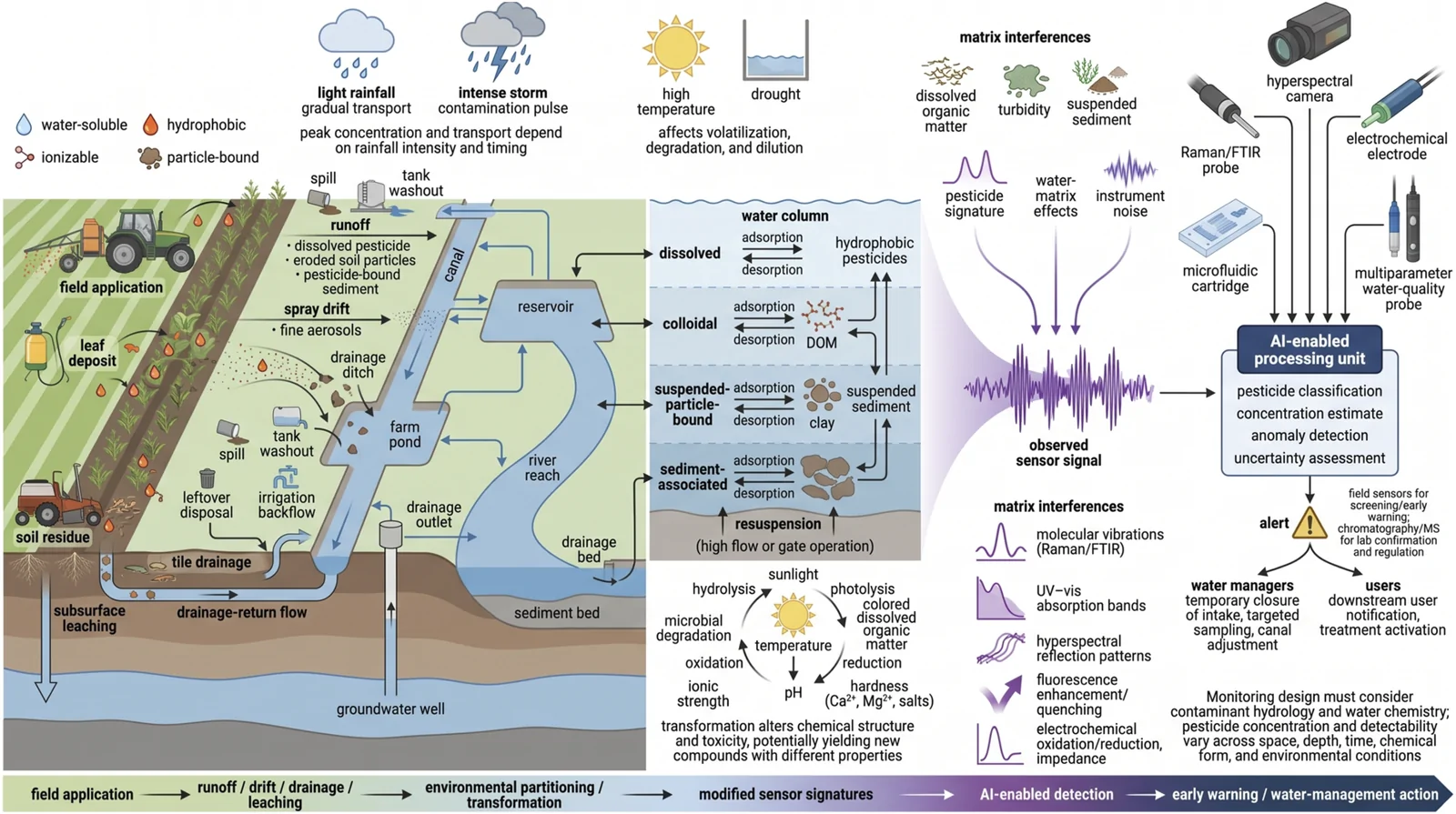
The conceptual source-to-decision framework linking pesticide entry and hydrological transport, environmental transformation and phase partitioning, matrix-conditioned sensor signatures, AI-assisted signal interpretation, and early-warning decisions in agricultural water systems. https://app.biorender.com/illustrations/6a73d9f158cea5cf0f4433e3.

Climate change modifies pesticide contamination through coupled changes in pesticide use, environmental transformation, and hydrological transport. Altered temperature regimes can affect volatilization, hydrolysis, microbial degradation, and pesticide partitioning, whereas changes in rainfall amount, intensity, and seasonality modify the relative importance of surface runoff, preferential flow, drainage, and groundwater recharge. Consequently, the receiving water's exposure to pesticides may shift from relatively diffuse background contamination toward short-duration concentration pulses associated with hydrological events.^5,6^ Extreme rainfall is particularly important when it occurs shortly after pesticide application, because residues that have not yet degraded or become strongly sorbed can be rapidly transferred from agricultural fields to canals, streams, reservoirs, and drainage networks. On the other hand, extended periods of dry weather may lead to changes in degradation and soil storage processes, and subsequent rainfall events can cause the re-release of soil-stored parent pesticides and transformation products.^7^ These climate-sensitive processes also cause changes in the temporal composition of contamination. At the catchment scale, the modelling of S-metolachlor and its transformation products has demonstrated that the concentrations of pesticides exported to rivers are significantly affected by both rainfall amount and intensity; a lot of rain results in higher concentrations of pesticides in rivers and a high intensity of rainfall over a short period of time leads to peaks in concentration due to increased overland flow.^8^ Pest and disease pressure changes due to climate change can also affect when, how often and what types of pesticides are used, adding to temporal variability in contaminant loading potential.^6^ Therefore, climate change is not only likely to lead to higher or lower concentrations of pesticides in water but also to higher concentrations occurring more frequently or less frequently, and to greater chemical variation, making for the more challenging characterization of pesticide occurrence upon infrequent sampling.

This leads to time variability, which results in a particular monitoring problem. Grab sampling and laboratory analysis can give very precise pesticide identification, but fixed time sampling can fail to detect pulses of short duration between sampling dates. The higher frequency or event-triggered measurement of parameters is thus needed for climate-responsive surveillance, alongside monitoring rainfall, discharge, temperature, turbidity, conductivity and other environmental parameters. In this setting, AI is helpful not because of the need for AI in the context of climate change, but because climate-sensitive pesticide signals are becoming more and more multivariate, nonlinear and non-stationary. Machine-learning models can integrate continuous sensor and hydrometeorological data, distinguish abnormal signals from changing environmental baselines, identify contamination events, and support the short-term forecasting of elevated-risk periods.^9^ Laboratory chromatography and mass spectrometry nevertheless remain essential for confirmatory identification and reference measurements. Climate change therefore strengthens the case for a complementary monitoring architecture in which continuous sensing and AI-assisted interpretation provide temporal resolution and early warning, while conventional laboratory methods provide analytical confirmation.

### 1.1 Limitations of conventional detection and the need for autonomous AI-driven water monitoring

Conventional methods used in the laboratory, such as gas chromatography, liquid chromatography, and mass spectrometry, are highly sensitive and accurate in terms of detecting pesticides. However, they are not very suitable for continuous, large-scale monitoring. These techniques are labor-intensive and costly and involve complex techniques for the sampling and preparation of samples.^10^ They also rely on skilled personnel, which leads to huge time delays between sampling and analysis. As a result, short-term contamination events (such as runoff pulses following rain) are often not detected. Spatial coverage is also limited and particularly difficult in big or remote irrigation systems.^11^ Artificial intelligence can be useful if the response of the sensors is multivariate, if it is nonlinear, if the sensors are subject to matrix interference, if the sensor drift is significant, or when the response needs to be integrated over various times, locations, and sensing modalities; however, when a selective sensor and conventional calibration or chemometric analysis can to remove interference affecting the target analyte, it is not necessarily superior. The benefits of autonomous monitoring platforms can therefore be enhanced, though the extent to which these platforms can be useful is dependent on the reliability of sensing and predictive models in response to realistic water chemistry and hydrological conditions.^12^ The main question of this review is then as follows: when can AI be usefully applied, to what extent, and under which types of pesticide chemistry, matrix complexity, sensing modality and deployment setting does it offer clear benefits for pesticide surveillance over conventional calibration, chemometric, and statistical approaches?

### 1.2 The scope, conceptual framework, and distinctive contribution of this review

This review is specifically focused on pesticide-residue monitoring in agricultural water systems (agricultural drainage water, farm ponds, reservoirs and rivers receiving agricultural runoff, and groundwater used for irrigation). Sensing involving food, crop surfaces, or general water quality is included only if it gives evidence for a principle that can be applied to the analysis of pesticides in water. The review therefore does not consider general water-quality prediction, crop imaging and pesticide-residue analysis in harvested commodities as direct pesticide sensing in agricultural water. Rather, it considers the environmental interactions and properties of pesticides in terms of the chemical identity that affects the signal available to a sensor, and then the computational analysis needed for reliable detection. The review is organized around the following six phases of the source-to-decision paradigm: (i) pesticide release and hydrological transport to agricultural water systems; (ii) structurally dependent ionization, degradation, sorption and phase partitioning, which determine the form of the pesticide available for measurement; (iii) the conversion of that chemical information into optical, spectroscopic, electrochemical, biosensor, or microfluidic responses; (iv) preprocessing, feature extraction, multimodal fusion, and AI-assisted classification or concentration estimation; (v) validation under conditions of realistic matrix interference, sensor drift, environmental domain shift, and external datasets; and (vi) operational deployment through fixed sensor networks, edge devices, autonomous platforms, and decision-support systems. This sequence does not separate out the various technologies relating to pesticide chemistry, environmental transport, sensing physics, artificial intelligence, and pesticide-water management decision-making, but rather connects them together as part of a whole.

This field has mostly been studied from a number of different perspectives recently. Pesticide-residue detection involves the use of multiple analytical techniques, which have been discussed in various papers, including those featuring spectroscopy, chromatography, biosensors, nanomaterials, and advanced analytical methods that combine analytical and AI tools or that are multimodal,^13,14^ often with a significant focus on food and crop matrices. While recent water-quality monitoring reviews focused on general physicochemical water-quality parameters, monitoring networks, and general water-quality prediction frameworks, they did not focus much on pesticide-specific chemical sensing.^9,15^ The present review aims at closing this gap by first discussing the impact of pesticide chemistry and hydrological fate on the measurable sensor response, the analytical advantages of AI in the processing of matrix-dependent or multimodal signals, and, to date, the validity of this advantage when moving from controlled experiments to heterogeneous agricultural-water environments. In this respect, the special contribution of this review is four-fold.

First, it connects the physicochemical behaviour and environmental fate of pesticides straight to their sensing, instead of talking about sensing technologies without reference to the analytes or water matrix. Second, it compares sensing methods based on monitoring-relevant parameters, such as selectivity, matrix tolerance, response time, calibration needs and/or field applicability. Third, it separates experimental AI-based pesticide detection from computational methods, which are largely prospective for this use. Fourth, it views external validation, uncertainty, sensor drift, domain transferability, and field deployment as part of monitoring performance, instead of as secondary implementation concerns. The resulting framework is intended to support the selection of an appropriate combination of pesticide target, sensing modality, analytical model, and deployment strategy to meet a defined agricultural-water monitoring objective. Fig. 1 summarizes the environmental and analytical front end of this framework by showing how pesticide sources, hydrological transport, transformation, phase partitioning, and water-matrix effects ultimately determine the signals presented to sensing and AI-assisted detection systems.

## 2 Pesticide chemistry, environmental speciation, and consequences for sensor response

### 2.1 Molecular structures, speciation, and phase distributions of pesticides in agricultural water

Pesticide detectability in agricultural water cannot be inferred from the application rate or total environmental concentration alone because the signal presented to a sensor is controlled by the molecular structure and physicochemical form of the analyte. Hydrophobicity, aqueous solubility, acid–base functionality, molecular charge, volatility, and affinity for organic matter determine whether a pesticide occurs predominantly as a freely dissolved molecule, an ionized species, an organic-matter-associated fraction, or a particle-bound contaminant. These differences are especially significant between classes of pesticides. Many organochlorines, like hydrophobic molecules, tend to bind more strongly to suspended particles, sediments and organic matter, while comparatively polar neonicotinoids are more likely to be highly mobile in the dissolved phase. Thus, the variety of physicochemical properties of pesticides currently in use has an impact not only on environmental transport, but also on analyte enrichment, extraction, separation, and direct accessibility by sensors.^16,17^ Ionizable pesticides should be considered separately since their apparent hydrophobicity, sorption, mobility and interactions with sensors are dependent on the pH relative to their acid–base dissociation behavior. For example, weakly acidic herbicides may exist in neutral and anionic form in varying proportions at different pH values, while protonatable compounds may be neutral or positively charged depending on the pH. Ionization alters electrostatic interaction with other recognition interfaces, such as dissolved organic matter, mineral surfaces, electrode coatings and molecularly imprinted polymers. Thus, the calibration activity at one laboratory pH may not match the calibration response in field water with another pH and/or ionic composition. Thus, the analytical target should be clearly specified as the total exposure (pesticide concentration), the freely dissolved parent compound or a specific chemical species, since these are not necessarily identical.^18^ A molecule with a chromophore may be detectable by UV-visible or fluorescence measurement, whereas bond vibrations can give Raman or infrared fingerprints. The electroactive functional groups might be oxidized or reduced at the surfaces of electrodes, and the pesticide could be bound to a biosensor using enzyme inhibition, antibodies, aptamers or other affinity interactions. In the case of electrochemical sensing, the structure and properties of the pesticide and the electrode material, as well as the composition of the electrolyte and the pH of the solution, all play a role in determining the electron-transfer kinetics, adsorption at the sensing interface, peak potential and current intensity. Therefore, a detection limit obtained with one sensor-pesticide combination cannot be extrapolated to another pesticide simply because both are of the same pesticide class.^19^

### 2.2 Degradation, transformation products, and matrix-dependent sensor response

Once a pesticide is introduced into agricultural water, its chemical identity may change at the sensor. The parent active ingredient can transform to one or more pesticide transformation products (PTPs) through hydrolysis, photolysis, oxidation–reduction reactions and microbial metabolism. Not all PTPs are necessarily detected analytically or totally removed after transformation; some may be mobile and persistent, or have different physicochemical and toxicological properties from their parent compounds.^20^ This is especially important for sensing technologies trained or calibrated on pure parent compounds. Absorbance in the UV-visible region can be lost, gained or modified; Raman or infrared bands can be changed and modified; fluorescence properties can be altered; redox properties can be altered, and metabolites can lose or gain affinity for the enzyme, antibody, aptamer, or molecularly-imprinted recognition elements. A sensor could then either underestimate or overestimate the overall chemical load from pesticides if it only reacts to the parent compound or if partially overlapping responses are produced by the transformation products.

The other fraction of the pesticide that reaches the sensing interface is dependent on the matrix composition. Dissolved organic matter (DOM), specifically humic and fulvic fractions, can complex with hydrophobic pesticides, affecting their freely dissolved concentration, as well as providing a broad background of optical absorption and fluorescence. Strongly sorbing pesticides can attach to suspended mineral and organic particles, and these suspended particles can also scatter incident radiation, contribute to turbidity and alter the Raman, fluorescence, UV-visible, and hyperspectral signals. Hence, the concentration recorded by an optical sensor can be the result of pesticide-specific absorption or emission, DOM background and/or particle scattering, algal pigments, and other dissolved components, rather than being a unique molecular fingerprint of a pesticide.^21^ There are different but overlapping matrix processes that have an impact on electrochemical and affinity-based sensing. Changes in ionic strength affect solution conductivity and electrostatic interactions at electrode and recognition surfaces, while pH can affect the ionization of pesticides, the kinetics of their reactions at the electrodes, and the activity of biomolecules; Ca^2+^ and Mg^2+^, which are associated with water hardness, can affect complexation, surface charge, and surface interactions. Exposed sensor surfaces can also be fouled by natural organic matter, suspended solids, microorganisms, and extracellular material that may progressively adsorb to the surface, affecting mass transfer, blocking recognition sites, increasing the sensor's impedance or causing baseline drift. These effects prevent the transfer of previously determined apparent sensitivity or selectivity from buffer or ultrapure water to irrigation canals, drainage water, reservoirs or groundwater. The physicochemical properties of the pesticide and the composition of the environmental matrix should therefore be considered as part of the sensing mechanism, instead of the experimental noise from outside.^17,19^ Analytically, there are three possible consequences of matrix effects: suppression of a true pesticide response, enhancement of a response, and the creation of an interfering signal that mimics a pesticide response. The implications of these outcomes are different for AI-assisted sensing. However, algorithms can learn to compensate for reproducible multivariate interference if the calibration datasets intentionally contain pH, turbidity, DOM, ionic strength, and co-contaminants within a range pertinent to the specific pesticide under analysis; this is not possible when the sensor does not record chemical information that is specific to the pesticide. It is thus essential to have chemistry-aware calibration prior to meaningful machine learning. Laboratory training with pesticide-spiked water can lead to the teaching of concentration-dependent patterns, which are not applicable in the field in the event of varying pesticide speciation, degradation products, and matrix compositions.

### 2.3 Spectral signatures, toxicological thresholds, and the regulatory context for automated pesticide sensing

The preceding chemistry has direct consequences for the interpretation of pesticide sensor signals: the analytical signature is a property of the chemical species interacting with the sensing interface under particular matrix conditions, rather than being an immutable property of the nominal pesticide. Spectral assignments measured for a pure standard can shift in intensity, broaden, overlap with matrix features, or disappear when the pesticide is ionized, transformed, sorbed to particles, associated with DOM, or present below the sensor's effective concentration range.^17,20^ Similarly, an electrochemical response depends on the electroactive form reaching the electrode, whereas a biosensor response depends on the preservation of the molecular feature recognized by the biological or synthetic receptor. Accordingly, spectral libraries and AI training datasets intended for environmental monitoring should include chemically plausible species, transformation products where relevant, and representative water matrices, rather than relying exclusively on pure pesticide standards.^19^ Pesticide chemical structures can be detected using powerful sensing devices by taking the optical, vibrational and electronic signatures of these structures. IR spectroscopy quantifies most of the most important vibrational modes associated with functional groups, including phosphate esters, nitro groups, carbonyls and aromatic rings. Raman spectroscopy offers an alternative source of data, particularly when a compound has symmetrical structures that have high-intensity Raman bands.^22^ Ultraviolet-visible spectroscopy determines electronic variations of the chromophoric content of pesticides; it is a quick detection technology but not necessarily specific. Hyperspectral imaging builds on the features of spectroscopy and digital imaging by acquiring continuous reflected spectra at hundreds of wavelengths. This allows machine learning algorithms to capture minor variations between water that is contaminated with pesticides and pesticide-free water. Fluorescence sensors are developed with the strength to exploit the natural or induced fluorescence of pesticide molecules or their by-products.^23^ All these spectral signatures are used in automated classification, anomaly detection, and the estimation of concentrations. They can therefore be used to construct AI-monitored systems.^24^

Analytical performance must therefore be evaluated against the chemical form of the pesticide and the matrix in which detection is required. Laboratory sensitivity alone is insufficient, because selectivity, matrix tolerance, response time, and validation in natural water determine whether a sensing platform can support operational surveillance. For example, reflectometric atrazine sensing achieved a detection limit of 0.35 µg L^−1^ and 15 min analysis but showed substantial humic-acid interference and cross-reactivity with related triazines in natural water samples.^25^ By comparison, second-order calibration led to the determination of imidacloprid even in the presence of other fluorescent pesticides and unexpected (and unanticipated) fluorophores, an example of how multivariate analysis can be useful in cases where chemical interference cannot be eliminated instrumentally.^26^Table 1 thus compares different sensing approaches not only on the basis of their sensitivity but also in terms of other criteria for monitoring.

**Table 1:** A systematic comparison of representative pesticide-sensing approaches relevant to agricultural-water monitoring

Sensing platform and principle	Target pesticide and verified analytical signal	Detection range/LOD	Selectivity	Matrix tolerance/interference	Response time	Validation level/sample context	AI/chemometric compatibility^a^	Reference
FIA-FTIR; absorbance-based vibrational detection	Malathion; quantitative FTIR chemigrams integrated over 1027–1017 cm^−1^	LOD: 12 µg mL^−1^	Compound-specific spectral region after extraction	Demonstrated in commercial formulations; direct environmental-water tolerance not established	NR	Laboratory validation; commercial pesticide formulations; no environmental-water validation	High for multiband spectral modelling, but the original analysis used conventional calibration	27
ATR-FTIR; vibrational spectroscopy	Glyphosate; pH-dependent bands assigned within the range of 1800–850 cm^−1^	Not developed as a LOD-based field sensor	Molecular vibrational information, but strongly species-dependent	Explicitly affected by pH and ionic strength; measurements performed in aqueous NaCl	NR	Controlled aqueous-system study; no sensor-level field validation	High for chemometric speciation analysis; field applicability requires matrix-specific calibration	28
Reflectometric interference spectroscopy; affinity-based optical sensing	Atrazine	LOD: 0.35 µg L^−1^	Cross-reactivity with simazine and terbutylazine limits compound-specific quantification	Strong interference from humic acids was observed	15 min	Authentic surface-, ground-, and lysimetric-water samples; *ex situ* analysis	Moderate–high; multivariate correction could model cross-reactivity and matrix effects	25
Photochemically induced fluorescence-FIA	Imidacloprid; photoproduct: *λ*_ex_ 334 nm, *λ*_em_ 377 nm	1–60 ng mL^−1^; LOD: 0.3 ng mL^−1^	Photochemical conversion improves detectability	Applied to natural-water samples with recovery of around 100%	Rapid flow-injection analysis	Natural-water samples; laboratory/*ex situ* analysis	Moderate; single-response analysis can be extended to multivariate interference correction	29
PIF excitation–emission matrices + second-order calibration	Imidacloprid	LOD: approximately 1 ng mL^−1^	Resolves imidacloprid in the presence of other fluorescent pesticides and unexpected fluorophores	Designed specifically to handle complex fluorescence backgrounds	NR	Real water samples with matrix interferents; laboratory/*ex situ* analysis	High; PARAFAC and U-PLS/RBL were directly demonstrated	26
Molecularly imprinted fluorescent sensor	Imidacloprid; fluorescence quenching	1–1000 nM; LOD: 0.3 nM	Molecular imprinting produced a specific response to imidacloprid	Tested in spiked environmental water and food matrices	≤1 min fluorescence response	Spiked environmental-water validation; no *in situ* deployment	High for multivariate fluorescence classification and drift/interference correction; AI not used in the original study	30
AChE electrochemical biosensor; inhibition measured by SWV	Chlorpyrifos	25–1000 nM; LOD: 2.2 nM	Sensitive to chlorpyrifos, but AChE inhibition is intrinsically class-responsive to organophosphates rather than uniquely molecular-specific	Evaluated with water samples and potential interferents	NR	Real matrix/spiked water and juice testing; no continuous field deployment	High for voltammetric feature extraction, interference correction and temporal drift modelling; AI not demonstrated originally	31
AuNP/graphene screen-printed electrochemical sensor	Glyphosate; indirect electrochemical response	LOD: 0.03 ppb	Selectivity demonstrated against phosmet, chlorpyrifos and glufosinate-ammonium	Similar LOD in buffer and surface-water matrix; recoveries: 93.8–98.9%	NR	Surface-water validation; portable format but *ex situ* measurement	Moderate–high for multivariate voltammetric analysis and sensor-drift correction; AI not used originally	32
SERS using Ag decahedral nanoparticles	Thiram; molecular Raman fingerprint	LOD: 1.0 × 10^−11^ M in aqueous solution	High chemical fingerprinting potential, but performance depends strongly on analyte adsorption and substrate reproducibility	Demonstrated in aqueous solution; complex natural-water interference not established	NR	Laboratory aqueous solution and fruit-surface validation; no environmental-water field deployment	High because full Raman spectra permit classification, regression and mixture deconvolution; AI not demonstrated originally	33

a AI/chemometric compatibility denotes the suitability of the resulting data structure for multivariate or machine-learning analysis; it does not imply that AI was experimentally used in the original study unless explicitly stated. NR = not reported in the cited primary study.

## 3 Advanced sensing technologies for irrigation-water pesticides

### 3.1 Comparative performance of optical-, spectroscopic-, and fluorescence-based pesticide sensing

The biggest advantage of vibrational spectroscopy is the molecular information it provides, with the sensitivity and matrix tolerance varying significantly from configuration to configuration. Although chemically interpretable bands can be obtained by conventional FTIR, they are gathered inside a spectrometer and not in the field during the continuous monitoring of processes, as was done for malathion in the analytical interval of 1027–1017 cm^−1^ and for glyphosate in the pH-sensitive interval of 1800–850 cm^−1^.^27,28^ Plasmonic enhancement can reduce the detection limit significantly, such as the limit of 1.0 × 10^−11^ M for the detection of thiram in an aqueous solution with silver decahedral nanoparticles.^33^ However, the SERS performance is dependent on the adsorption chemistry, distribution of hotspots, reproducibility of the substrate and matrix competition, and an extremely low LOD in the laboratory may not guarantee reliable environmental-water performance.

There are platforms that offer a different sensitivity/selectivity balance, using fluorescence techniques. In the case of imidacloprid, the use of maximum excitation and emission wavelengths (334 nm and 377 nm, respectively) allowed the detection of imidacloprid at 0.3 ng mL^−1^ in water.^29^ Molecular imprinting was then validated, with a LOD of 0.3 nM and a response time of 1 min, but this was not directly deployed in the field and was instead used to test spiked environmental matrices.^30^ Perhaps most importantly, using high-dimensional fluorescence data, PIF excitation–emission matrices and PARAFAC and U-PLS/RBL were successful in quantifying imidacloprid in real water samples, even in the presence of other fluorescent pesticides and unknown fluorophores.^26^ Thus, platform selection should depend on whether the monitoring priority is molecular specificity, ultratrace sensitivity, rapid response, resistance to matrix interference, or compatibility with multivariate analysis, rather than on LOD alone.

### 3.2 Electrochemical and microfluidic biosensing platforms for smart pesticide monitoring

Electrochemical sensors are compact and low-power devices that can be used to detect pesticides in the field. They operate by converting chemical or biochemical interactions at the electrode surface into measurable electrical signals. Common configurations include amperometric, voltammetric, and impedimetric sensors. Enzyme-based designs include cholinesterase inhibition for the specific targeting of organophosphate insecticides.^34,35^ Sensor performance can be enhanced by incorporating nanomaterials such as graphene, carbon nanotubes, and metallic nanoparticles, which can increase the active surface area and facilitate electron transfer. These materials increase the surface area, increase the speed of electron transfer, and increase the selectivity. As such, these sensors are applicable for permanent deployment in autonomous water-monitoring platforms.^36^ Microfluidic lab-on-chip biosensors allow further shrinking and combine sampling, reaction, and detection into a portable device. These systems require small amounts of reagents and highly specific recognition elements; they have enabled the rapid, reproducible and multiplexed detection of pesticides. Aptamer- and peptide-based platforms further illustrate the versatility of selective molecular recognition for compact sensing architectures.^37,38^ They can be linked to irrigation canals directly or to robotic samplers. Embedded electronics, wireless communications and AI-driven analysis of data allow for the observation of trends and detection of anomalies, supporting decision-making in real time. The combination of these two platforms makes electrochemical and microfluidic biosensors a crucial element of smart agricultural water quality monitoring networks.^39^

Electrochemical platforms generally offer stronger prospects for portable and low-power deployment than benchtop spectroscopic systems, but their selectivity depends on the recognition mechanism. AChE inhibition produced a chlorpyrifos LOD of 2.2 nM using a screen-printed biosensor, although enzyme inhibition is fundamentally responsive to organophosphate activity and therefore does not automatically provide molecular-level selectivity among structurally related inhibitors.^31^ In contrast, an AuNP/graphene screen-printed sensor for glyphosate achieved a LOD of 0.03 ppb in both buffer and surface water and recoveries of 93.8–98.9%, with selectivity tested against several other pesticides.^32^ These examples show why sensitivity, recognition specificity and matrix validation must be evaluated together before a platform is considered suitable for pesticide-specific field surveillance.

### 3.3 Multi-sensor fusion, drift management, and calibration in smart water monitoring

No single sensor can be used to capture the complex dynamics of pesticides in irrigation systems. Multi-sensor fusion architectures are thus of the utmost importance. The combination of complementary modalities—hyperspectral imaging, electrochemical sensors, fluorescence probes and microfluidic biosensors—in fused systems can provide more robust and reliable assessments than any single sensor. Data from fixed stations, mobile platforms and remote sensors could be combined at the feature or decision level, leveraging machine learning frameworks. This increases the accuracy of detection, decreases false positives and enables cross-validation under different environmental conditions.^40^ Edge computing goes further to improve these architectures by supporting decentralized processing, reducing the number of communications, and improving response times. Long-term assignments, particularly field deployment, must incorporate drift and noise from the evolution of the material as a result of material aging, fouling, and environmental changes, which can reduce accuracy if they remain uncorrected. Effective calibration protocols (*e.g.* periodic reference measurements, baseline correction and normalization) are thus important. Long-term fusion systems therefore require periodic reference measurements, modality-specific normalization, cross-calibration, and the explicit tracking of sensor drift. These requirements become particularly important when optical and electrochemical measurements are combined because each modality responds differently to matrix composition and sensor ageing.^14,41^ The computational treatment of these matrix-dependent effects and cross-modal signals is discussed in Section 5.4. The relationships among complementary sensing modalities, environmental interference, calibration requirements, and cross-modal data fusion are summarized in Fig. 2.

**Fig. 2:**
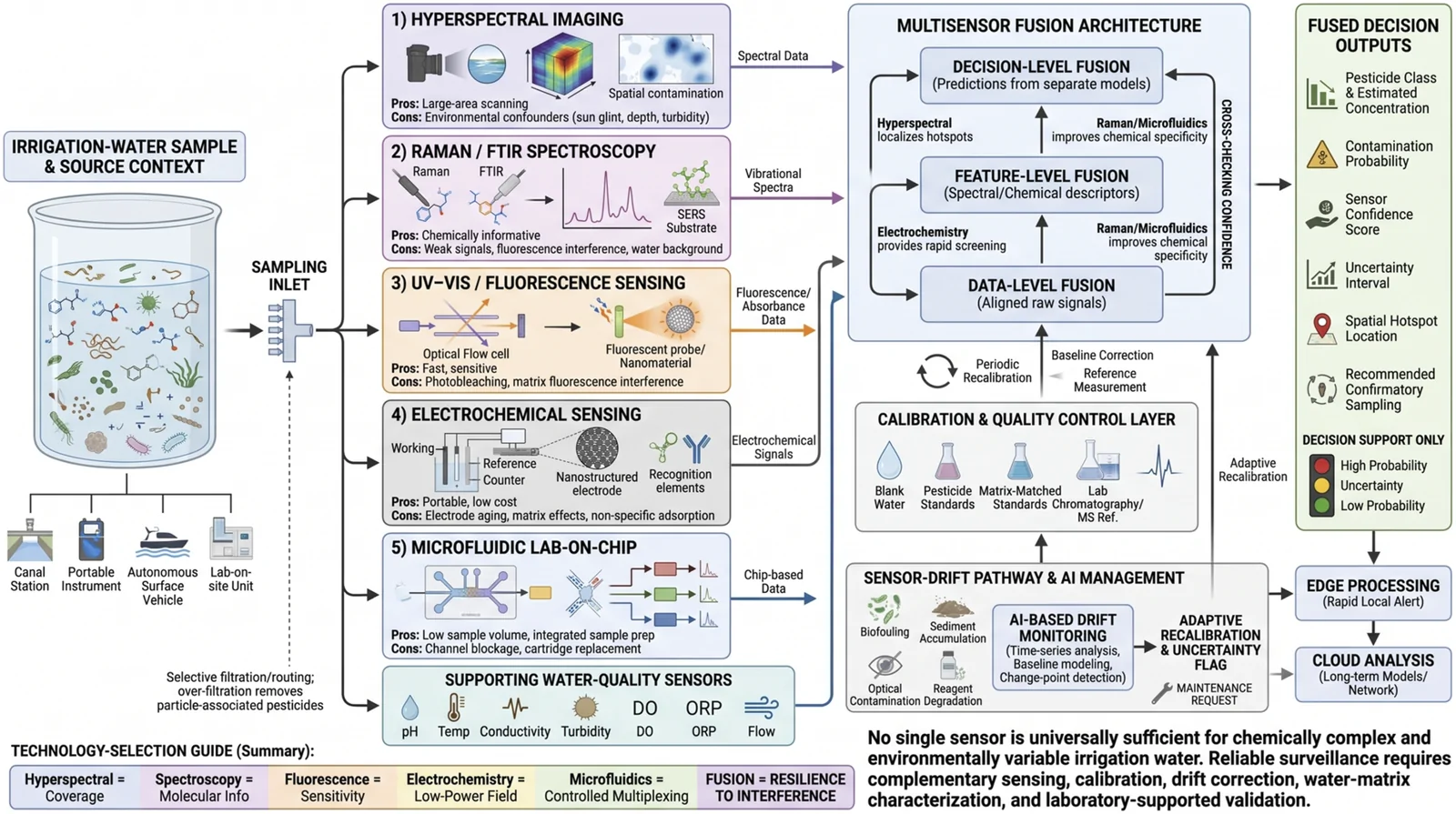
Complementary pesticide-sensing platforms, environmental interference sources, calibration requirements, and multimodal data-fusion architecture. https://app.biorender.com/illustrations/6a73db31472f128943c5488e.

## 4 Data acquisition and preprocessing pipelines

### 4.1 Radiometric, atmospheric, and water-surface corrections for reliable spectral analysis

Radiometric calibration is the first necessary process used to convert raw digital numbers into physically interpretable radiance or reflectance values. It compensates for instrument-response effects, including any dark signal, detector non-uniformity, and calibration-coefficient variability, thereby improving comparability among measurements acquired at different times or by different sensors.^42^ Airborne or satellite data are also very important in atmospheric correction. The spectral signatures that are transmitted to the sensor are modified by scattering and absorption by gases, aerosols and water vapor. Radiative transfer models of the atmosphere like MODTRAN or empirical line correction estimate the atmospheric path radiance and transmittance and then eliminate them. Atmospheric correction is still needed to record reflectance, even in the case of the monitoring of irrigation water by near-surface platforms, especially with hyperspectral drones or high-altitude photography.^43^ Aquatic correction corrects distortions that are unique to water, such as the uptake of water molecules, backscatter by suspended solids and the effects of colored dissolved organic matter. The optical characteristics of water change dramatically with wavelength, particularly in the near infrared range, at which point there is a high level of absorption. Correction models remove the intrinsic optical characteristics by removing the effects of a surface wave, scattering layer and bottom reflectance where possible.^44^ Determining albedo on the bottom and the depth of water in shallow irrigation canals is complex and thus algorithms need to consider mixed spectra due to substrate reflectance. The combination of these calibration processes provides a clean and consistent basis for the later downstream processing and machine-learning classification of pesticide contamination.^45^

Optical measurements are significant when retrieving the correct water surface reflectance. In spectra, bright spikes that are uninformative are produced due to specular reflection, or sun glint. The spikes are particularly troublesome in near-infrared spectra, where they have the ability to obscure fine spectral variations due to traces of pesticides. To allow effective analysis, glint must be removed.^46^ A number of glint-removal methods, including the use of polarization filters, empirical line subtraction, and the spectral band ratio, are available. One of the methods used takes advantage of the high correlation between the visible and near-infrared channels in pixels affected by glint. The approach detects near-infrared values and conserves the water reflectance by regressing near-infrared values onto visible bands, thus eliminating the glint component.^47^ In addition to glint, water surface reflectance retrieval should take into account the effects of anisotropy caused by waves and viewing geometry. Such factors change the water surface bidirectional reflectance distribution function (BRDF). The importance of illumination-view geometry for quantitative reflectance retrieval is also well established for spectroscopic applications.^48^ The measured spectra vary with the sun angle, wind speed and sensor orientation. Meteorological information and radiative-transfer simulations can be used to correct angular dependencies when using correction models .^49^ Precise reflectance recovery is especially noteworthy in cases where pesticide detection is based on weak spectral features or a machine learning model that is vulnerable to minor discrepancies. These corrections can enhance the accuracy of classification models by making the reflectance signal more stable, and they allow the making of comparisons over time series across different environmental conditions.^50^

### 4.2 Spectral unmixing and noise reduction for robust pesticide signal extraction

Spectral measurements of natural irrigation water involve mixtures, not single substances. Therefore, before analysis, we have to separate the components (unmix) and remove the noise. Unmixing algorithms separate the mixed signal into endmembers and fractional amounts and allow us to separate pesticide-related signals. Linear unmixing is based on the assumption of linear mixing, and it is quick for shallow or moderately turbid water. Unsupervised techniques such as VCA and N-FINDR are able to learn endmembers directly from the data and thus are able to detect very small contamination signals without prior knowledge.^51^ Noise removal helps to improve the quality of features, especially poor pesticide signatures. Savitzky–Golay filtering can be used to smooth spectra without the loss of peak shapes and derivatives. Wavelet denoising eliminates high-frequency noise while retaining low-level spectral information. Applying proper unmixing and denoising steps reduces the uncertainty and improves the accuracy and stability of machine learning models that are used to monitor pesticides.^52^

### 4.3 Feature engineering, normalization, and synthetic data strategies for AI-based pesticide monitoring

Spectral and hyperspectral datasets for pesticide monitoring are large in size and highly correlated; therefore, the reduction of features is necessary for faster computation and improved model performance. Linear methods, such as principal component analysis, eliminate redundancy and noise by projecting the data onto orthogonal components. The components of the overlap of the spectral sources can be separated by independent component analysis. Nonlinear manifold-learning techniques, including t-SNE and UMAP, preserve the local neighborhood structure and reveal hidden contamination patterns, which will help in clustering and visualization during complex studies on irrigation and water.^53^ Multi-view uncertainty-based feature selection and semi-supervised knowledge distillation provide additional strategies for high-dimensional, label-limited datasets.^54,55^ Large monitoring networks must also normalize data from different sensors and align spectra to ensure that the results are consistent regardless of platform. Variations in the type of sensor, dynamic range and wavelength response can render datasets incompatible. Standard methods for scaling and alignment account for baseline shifts and wavelength mismatches, which opens the door to general machine learning models, which can be applied across sites, seasons and sensor systems.^56^ Another problem in AI pesticide detection is the scarcity of labeled training data or data for low concentrations of pesticides or new compounds. Synthetic datasets, which are created by chemical and radiative-transfer simulations, bridge this gap, in which increasingly realistic spectra are produced under different environmental conditions. Augmenting data by injecting noise and mixing scenarios further helps make training robust and increases model robustness. If there is a lack of real data, using synthetic data and transfer learning accelerates the development of robust and scalable systems for monitoring pesticides.^14^

Preprocessing and quantitative interpretation do not inherently require AI. For spectra obtained under controlled conditions, established approaches such as baseline correction, Savitzky–Golay filtering, principal-component analysis, and partial least-squares regression can reduce noise, compress correlated variables, and quantify analytes with comparatively low computational complexity.^52,57^ Second-order chemometric calibration has also successfully quantified imidacloprid in real water samples containing fluorescent interferents, demonstrating that complex pesticide signals can sometimes be resolved without machine-learning models.^26^ Accordingly, model complexity should be increased only when conventional calibration or chemometric methods fail to capture the dimensionality, nonlinearity, temporal variations, or matrix dependence of the sensor response.^15^

## 5 From conventional chemometrics to AI-assisted pesticide-sensor interpretation

### 5.1 When is AI actually necessary?

AI is most justified when the relationship between sensor output and pesticide concentration cannot be represented adequately by simple calibration or conventional multivariate statistics. Such situations include nonlinear responses caused by interacting matrix variables, high-dimensional hyperspectral or excitation–emission data, the simultaneous interpretation of several sensing modalities, and time-dependent signals affected by environmental variability or sensor drift.^9,14^ By contrast, a selective sensor producing a stable univariate response in a defined matrix may require only conventional calibration, while correlated spectral datasets with approximately linear concentration relationships can often be treated effectively using PCA or PLS-based chemometrics.^57^ The principal advantage of AI for rapid monitoring lies in automated interpretation rather than in accelerating the underlying chemical measurements. A machine-learning model cannot shorten an intrinsically slow recognition reaction, sample-extraction step, or sensor equilibration process; however, once the signal is acquired, trained models can perform automated classification, concentration estimation, anomaly detection, and the integration of multiple sensor variables without repeated manual analysis.^9^

Higher predictive accuracy should likewise be demonstrated rather than assumed. AI models should therefore be benchmarked against appropriate simpler baselines, including linear regression, PLS regression, conventional classification, or threshold-based detection, depending on the analytical task.^15^ If a simpler model provides comparable error, sensitivity, and external-validation performance, the additional computational complexity of deep or ensemble learning is not analytically justified. More complex AI becomes defensible when it produces reproducible gains under independent validation, particularly for nonlinear matrix correction, multimodal fusion, rare-event detection, or temporally varying contamination patterns.^9,14^

### 5.2 Bayesian and ensemble learning approaches for spectral pesticide detection

Bayesian and probabilistic models provide a good approach for managing uncertainty during spectral pesticide detection, which is a common problem with irrigation systems due to noise, mixed signals and varying environmental conditions. Bayesian classifiers attempt to determine the probability of a spectral signature being a member of a specific pesticide class based on a combination of the actual data and previous data and thus work well when spectra overlap or when spectra are ambiguous. Extensions, such as Gaussian mixture models, can handle multimodal spectral distributions, and Bayesian regression estimates pesticide concentrations with a quantification of uncertainty, which is helpful for decision-making in a risk-based way.^58^ Ensemble learning helps to increase the robustness of classification by combining multiple simple models into one big and powerful predictor. Methods such as random forest and gradient boosting are suitable for handling high-dimensional hyperspectral data and prevent overfitting. They also capture nonlinear relationships that often appear in cases of mixed contamination. They can be used for ranking useful spectral features, which improves their interpretability, and can be used to optimize the design of the sensor. Bayesian and ensemble approaches are therefore most useful when uncertainty, nonlinear interactions, or heterogeneous predictor variables cannot be handled adequately by simpler calibrated models; their use should be justified through comparison with appropriate statistical or chemometric baselines rather than by predictive accuracy alone.^9,15^

### 5.3 Kernel-based SVM methods and feature engineering for spectral pesticide analysis

Kernel-based support vector machine (SVM) methods are suitable for identifying pesticides in high-dimensional spectral data. They capture non-linear relationships between wavelength bands. By spectral transformation into a transformed feature space using kernels such as radial-basis functions and polynomial functions, SVMs allow the separation of slight pesticide-induced changes without a natural background signal. Spectral-structural SVM variants enhance the performance further by the addition of spatial context in addition to spectral features, which is a crucial advantage in the hyperspectral analysis of irrigation channels. As they are based on a small set of support vectors, these models are resistant to overfitting and work even in the absence of sufficient data to train them. Related regression forms are even used for the quantitative estimation of pesticide concentrations.^59^ Feature engineering is used to improve these models for the chemically meaningful inputs of raw spectra. Derivative spectra, band ratios and spectral indices enhance low levels of absorption and reduce environmental noise. Automated feature-selection methods are then applied to the most informative wavelengths, which reduces the dimensionality. Using knowledge of the chemical domain allows researchers to be sure that the selected features will not be random noise and will represent true molecular processes that increase the interpretability and computation efficiency of AI-driven pesticide monitoring systems.^60^

### 5.4 Managing data imbalance and ensuring cross-regional generalization in AI models

The uneven distribution of polluted and clean samples is one of the largest issues in pesticide detection. In actual irrigation water, pesticides are only infrequently observed; therefore, most measurements are of clean or background conditions. This skew may lead to the majority class being more likely to be represented by models, with the slight but critical contamination being overlooked. Examples of common fixes are oversampling, undersampling, and data augmentation. An example is the synthetic minority oversampling technique that generates novel contaminated spectra by mixing existing samples of minority species, which produces enriched data but does not alter the overall statistics.^61^ Another technique is cost-sensitive learning, which imposes more penalties on the incorrect classification of minority species during training. This motivates the model to pay attention to pesticide indicators. Other resampling methods, such as balanced random forest, also resample within the training loop. In the case of pesticide data scarcity, anomaly detection might come in handy, as the contamination is regarded as an outlier. These techniques can keep the recall of the model high with regard to contamination, which is critical for risk management and early alerts in agriculture.^62^

Environmental conditions vary broadly among irrigation networks, leading to significant problems in the application of models that were trained based on one region, season or sensor configuration. Agricultural-water modification and cross-regional pollution transfer further emphasize the spatial heterogeneity of environmental risk.^63,64^ Factors such as turbidity, dissolved organic matter, salinity, and bottom reflectance generate domain shifts that decrease model accuracy. This is addressed by domain adaptation methods that find a solution to differences in the feature distributions of both source and target data. These approaches include reducing maximum mean discrepancy, domain alignment in adversarial systems, and transfer learning to reduce the discrepancies between datasets by changing model parameters or feature spaces.^9^ Sample-migration strategies provide another approach for transferring information across temporally varying remote-sensing domains.^65^ Domain adaptation is of particular importance in pesticide monitoring, where the models are trained on synthetic or laboratory data that will be required to be applied in practice. Small local data fine-tuning enhances generalization at the cost of no longer having the benefits of large datasets. Multi-domain learning models also enhance resilience by training models on a variety of datasets at once so that they can learn integrated representations that reflect global patterns and also adapt to regional variations. To use AI-based monitoring systems on national or transboundary irrigation systems, cross-regional generalization is a necessary attribute to guarantee that monitoring will be performed consistently in diverse environmental settings.^66^

### 5.5 Matrix-aware AI correction and cross-modal sensor fusion

Environmental matrix effects are sensor-specific and not just generic “noise.” A variety of DOM components may bind pesticides and may also give rise to optical absorption or fluorescence backgrounds: humic substances, for instance, caused significant interference in reflectometric atrazine sensing in natural water.^25^ Apart from this, turbidity and suspended particles also scatter light and modify the apparent intensity of reflected light, absorbance, Raman, and fluorescence signals; pH and ionic strength can also affect ionization, adsorption, and electrostatic interactions between the pesticide and the sensing surface.^18,19^ The value of the analytical target can also be changed due to the environmental degradation of the pesticide, which may result in the formation of transformation products with properties different from the parent pesticide.^20^ Biofouling represents a type of interference that can vary with time, as microorganisms attach to sensors and form biofilms that affect sensor response and mass transport, and eventually reduce the accuracy of measurements.^67^ The explicit measurement of interfering variables during model calibration is thus the most defensible place for AI-based correction. It is possible to include other matrix parameters like pH, conductivity, turbidity, temperature, and optical DOM surrogates in the primary pesticide signal, thereby distinguishing matrix-associated variations from analyte-related variations, by using a matrix-aware model.^9^ Multivariate correction has already been proven to be useful for pesticide sensing, achieving imidacloprid detection in real water samples without considering other fluorescent pesticide contaminants and unexpected fluorophores *via* the use of excitation–emission fluorescence matrices and U-PLS/RBL and PARAFAC.^26^ However, if the signal is totally suppressed, there is serious fouling, or the selectivity of the sensor is not sufficient to measure the signal, then the chemical info cannot be recovered by AI training—the data has to be cleaned, recalibrated, or conditioned, or the sensor must use a different sensing principle.^67^

The introduction of more than one type of detection technique adds a further layer of complexity, as the effects of dimensionality, units, sampling frequency, detection limits, calibration behavior, and environmental interference on optical, electrochemical, fluorescence, and biosensor outputs are not the same. The four main challenges of fusion—temporal synchronization, normalization, missing data and cross-calibration—are present and require the preservation of modality-specific uncertainty^14^ to enhance trust in multimodal pesticide-detection frameworks. The integration of heterogeneous representations can be enhanced by dynamic–static alignment, cross-attention fusion and multilevel semantic alignment, as revealed by related cross-modal studies.^68–70^ Data-level fusion is suitable for signals that are synchronized and directly comparable, feature-level fusion is suitable when the spectra and electrochemical descriptors are different, and decision-level fusion is suitable when individual sensors need independent calibration or there are different sensors that produce fundamentally different signals. More recent electrochemical–optical pesticide-sensing analyses also highlight that multimodal fusion can minimize false positives, but this also adds complications to integration and demands strong real-sample validation.^41^ In line with this, cross-regional generalization should rely on blocked or external validation across independent environmental domains and not be made by random partitions of pooled datasets.^71,72^

## 6 Deep learning architectures: evidence maturity and transfer to pesticide-water monitoring

To distinguish demonstrated capability from technological extrapolation, the evidence discussed in this section is classified into three maturity levels: (i) experimentally demonstrated pesticide detection, even when tested in food or crop matrices rather than water; (ii) demonstrated application to general water-quality or contamination problems without pesticide-specific validation; and (iii) prospective application to pesticide monitoring inferred from performance in adjacent domains. This distinction is necessary because the successful implementation of an AI architecture for hyperspectral crop analysis or generic water-contamination modelling does not establish its validity for pesticide sensing in complex agricultural water samples.

### 6.1 CNN-based deep learning architectures for spectral–spatial pesticide detection

Convolutional neural networks (CNNs) have become critical in spectral-chemical analysis as they have the capabilities to extract hierarchical features from complex hyperspectral and multi-sensor data automatically. Pesticide signals are very weak and nonlinear and may be readily obscured by turbidity, dissolved organic material and sediment dispersal during irrigation water monitoring. Degradation-aware and cross-temporal feature-enhancement methods provide transferable strategies for recovering weak signals under conditions of noise and temporal variation.^73,74^ CNNs would be ideal for locating these weak patterns as they learn localized filters that identify typical spectral textures, absorption features and contextual relations between wavelengths and spatial dimensions.^75^ One-dimensional CNNs directly manipulate spectral vectors and are therefore highly efficient for use with point-based spectroscopy like Raman measurements and FTIR and fluorescence measurements. The models predict wavelength-sensitive correlations and the presence of peaks, and they are able to learn discriminative spectral patterns relating to the presence of pesticides. They do not require a high level of computation and are suitable to be incorporated into edge devices in autonomous surface vehicles (ASVs) or autonomous underwater vehicles (AUVs), or into microfluidic sensors.^76^

Two-dimensional CNNs view hyperspectral imagery as layers of spectral bands, and they can be used to extract spatial features, including contamination plumes, mixing patterns, and features of the water surface. Such models are able to differentiate between spectral changes that are related to pesticides and those produced by the environment, such as by algae or aggregates of sediments.^77^ Three-dimensional CNNs extend this ability because they analyze the spectral and spatial dimensions together. Many higher-order interactions between wavelength bands and local spatial structures can be modeled, as hyperspectral cubes are handled as volumetric data. The performance of classification using this holistic representation can be significantly enhanced in cases where pesticide distributions are spatially coherent, *e.g.* contamination hotspots along irrigation channels.^78^ Another advantage of CNNs is that transfer-learning models can utilize pretrained weights from large-scale spectral data or even natural images, which primarily accelerates convergence and fills feature representations. CNNs have also been one of the most formidable and popular architectures in pesticide detection due to their strength, scalability, and multimodality.^79^

Vanishing gradients are experienced in deeper layers by traditional CNNs that are effective at feature extraction. This is addressed by residual networks through skip connections, which allow gradient flow between layers. These lines allow the creation of rather deep models that learn some complex spectral patterns. CNNs used as residual images are more effective at pesticide detection than conventional designs; they maintain fine spectral information and nonlinear environmental interactions that are not reflected in shallow designs.^80^ This idea is the basis of dense networks, which connect one layer to every subsequent layer. This maximizes feature reuse and promotes more feature-rich representations. Dense blocks are effective with hyperspectral data, since this compels the model to introduce a combination of information across a large number of spectral scales, and hence the model becomes more sensitive to the weak organic signals associated with pesticide contamination. The designs are highly accurate with few parameters, which assists in implementation on resource-bound aquatic robots.^81^ Hybrid CNN-transformer models combine the convolutional merits with long-range attention features. Layers in CNNs capture local spectral information, and transformer blocks capture global interrelations between wavelength and space. This combination overcomes the difficulty of CNNs in modelling long-range interactions while reducing the large-data requirements of transformers. In monitoring irrigation water, hybrids are best used when there is a mixture of pesticides and more complicated environmental aesthetics that demand finer details, as well as a general view to accurately detect a pesticide.^82^

Advanced D-learning techniques under consideration include CNNs, most of which have direct experimental evidence for detecting pesticide residues; however, the number of validations is still limited, and most of them are not in aquatic matrices. Hyperspectral imaging with 1D-CNN successfully discriminated pesticide residues on the surface of Hami melons, which demonstrated that convolutional models can extract pesticide-related spectral features in a controlled crop-matrix environment.^83^ Although CNNs are experimentally proven for pesticide detection, their effectiveness in irrigation water cannot be deduced from these studies due to the different analytical constraints posed by the presence of dissolved organic matter, turbidity, speciation, and much lower concentrations of analytes in this matrix.

### 6.2 Transformers and graph neural networks: demonstrated applications and remaining pesticide-water gaps

Transformer architectures have now progressed beyond purely conceptual use in pesticide analysis, but direct evidence remains concentrated in food matrices. Hegde *et al.* developed SERSFormer-2.0, which combined surface-enhanced Raman spectroscopy with a transformer architecture for the simultaneous classification and concentration prediction of multiple pesticides in spinach and strawberry samples. The model achieved an accuracy of 0.999, an *F*1 score of 0.992, precision of 0.990, and a recall of 0.996 for mixed-pesticide analysis.^84^ This study demonstrates that transformer attention can resolve complex multicomponent pesticide spectra; however, this does not establish performance in agricultural water, where concentrations, matrix compositions, transformation products, and temporal sensor drift differ substantially from prepared food-surface samples. Transformers should therefore be classified as having been experimentally demonstrated for pesticide detection but not yet validated for routine pesticide surveillance in agricultural water systems.

Graph neural networks occupy a different maturity level. Their ability to represent connected water systems has been experimentally demonstrated for general contamination problems. Li *et al.* used a gated graph neural network incorporating network topology, flow direction, and spatiotemporal water-quality data to identify contamination sources in water-distribution systems, achieving 92.27% accuracy for narrowing the source to five candidate locations using 2 h of sensor data.^85^ GNN-based approaches have also been applied to general river-network water-quality prediction, supporting their suitability for use in spatially connected hydrological systems.^86^ However, these studies did not use pesticide-specific chemical-sensor signals or model pesticide transformation and partitioning. Thus, GNNs are demonstrated for network-scale water-contamination modelling but remain prospective for pesticide-specific sensing and transport reconstruction in agricultural-water networks.

### 6.3 Temporal forecasting and generative deep learning for pesticide monitoring

Pesticide levels in irrigation systems change very quickly. They are affected by rainfall, runoff, irrigation cycles and chemical degradation. It is impossible to represent these dynamics through static spatial or spectral models. Recurrent neural networks represent another branch of neural networks that are designed to model long-range temporal dependencies in time-series data, particularly long short-term memory (LSTM) networks and gated recurrent unit (GRU) networks. LSTMs are able to use gated memory mechanisms to record delayed and seasonal contamination patterns. GRUs offer comparable performance with fewer parameters, which supports their use in energy- and resource-limited monitoring platforms. Combined with continuous data streams from fixed stations and autonomous vehicles, these models make it possible to get early warning of spikes in contamination, and also to make proactive decisions in the management of water systems.^87^ Hybrid designs, including convolutional neural networks (CNNs), LSTMs, convolutional LSTMs or attention mechanisms that combine convolution and LSTM, are further useful for improving forecasting as they help to extract spatial features while learning temporal features. Generative deep learning models have complementary use with forecasting models, such as in anomaly detection and data augmentation. Autoencoders enable the determination of deviations in the considered spectral patterns by reconstructing expected background signals, thus enabling the early detection of unknown or emerging pesticides. Variational autoencoders enhance background modeling with probabilistic latent spaces and produce realistic synthetic spectra for training under data-scarce conditions. Generative adversarial networks can generate very realistic spectral signatures, including fake contamination scenarios, which enhances the robustness of models for the detection of pesticides in airborne acquisition systems, stress testing, and domain adaptation in AI-based pesticide monitoring systems.^88^

### 6.4 Self-supervised and physics-informed learning: current evidence maturity

Self-supervised learning is attractive where large volumes of unlabeled spectra are available, because representation learning can precede task-specific fine-tuning; however, its direct validation for pesticide sensing in agricultural water remains limited. Its role in the present context should therefore be considered prospective until models trained on unlabeled environmental-water spectra are externally validated against independently measured pesticide concentrations and representative matrix variability. More broadly, the hyperspectral deep-learning literature supports the potential of representation-learning approaches for reducing dependence on extensively labelled datasets, but transfer to pesticide-water sensing still requires experimental confirmation.^24^

Physics-informed neural networks have reached a higher maturity level in general water-contamination modelling, but not yet in pesticide-specific sensing. Duan *et al.* developed a hierarchical PINN for contamination prediction in a 40-pipe, 35-node water-distribution network and reported a test MAPE of 0.31%, demonstrating that physical constraints and transfer learning can improve contaminant prediction within networked water systems.^89^ Nevertheless, the model addressed generic contamination rather than pesticide-specific sorption, degradation, speciation, or sensor response. PINNs should therefore be classified as having been demonstrated for general water-contamination modelling but as prospective for pesticide-specific fate reconstruction and sensor-assisted forecasting. Their application to agricultural water samples will require governing equations and training data that explicitly represent pesticide advection, dispersion, transformation, phase partitioning, and measurement uncertainty.

### 6.5 Training design, external validation, and uncertainty in pesticide-sensing AI

The reliability of pesticide-sensing AI depends as strongly on dataset design as on model architecture. Neural-network approximation has also been used to compensate for unmodelled dynamics and external disturbances in uncertain systems.^90^ Training datasets should span relevant pesticide concentrations, uncontaminated controls, co-occurring chemicals, and representative variations in pH, dissolved organic matter, turbidity, ionic strength, temperature, and other matrix variables that affect sensor response.^9,24^ Repeated spectral scans, technical replicates, or measurements generated from the same prepared sample should not be treated as fully independent observations when dividing data because correlated observations distributed across training and test sets can inflate apparent predictive performance.^71^ Dataset size should therefore be considered alongside chemical diversity, matrix coverage, the independence of observations, class balance, and concentration coverage, rather than focusing only on the total number of spectra. Training–validation–test separation must reflect the intended deployment task. Random splitting is inadequate when observations share location, sampling date, preparation batch, sensor device, or other dependence structures, because information from nearly identical observations can enter both training and testing partitions.^71^ Environmental modelling studies have shown that random *k*-fold cross-validation can substantially overestimate transfer performance compared with leave-location-out or leave-time-out validation.^72^ For pesticide monitoring, model development should therefore use grouped or blocked partitions where appropriate, followed by a final external test set that is not used for feature selection, hyperparameter optimization, threshold selection, or model calibration. External validation should preferably involve a different water body, sampling campaign, matrix composition, sensor unit or manufacturing batch, and, where feasible, a different season.

The introduction of a pesticide signal into the environment is accompanied by a domain shift, as the distribution of the pesticide signal and interfering variables in natural water is different from the distribution of these variables in laboratory standards. Even if the concentration of the target pesticide does not change, differences in turbidity, DOM, pH, ionic strength, degradation products, co-contaminants, and temperature can cause a shift in feature distributions, which in turn can lead to a reduction in the accuracy of a model.^9,91^ Changes in baseline, sensitivity, response dynamics and inter-sensor reproducibility are sources of drift in the temporal domain that are well known in multivariate chemical-sensing systems.^92^ The sensing interface may also be altered by fouling and ageing, which can occur over the longer term during environmental deployment.^67^ These effects can be minimized by domain adaptation, recalibration or transfer learning, but success must be tested based on real post-transfer data and not on the data used for adaptation. Point estimates should be accompanied by prediction uncertainty when AI generates an environmental alert or management decision. Ensembles and related uncertainty-quantification methods, along with Bayesian approaches, can, to a variable extent, separate uncertainty due to noisy observations from uncertainty due to limited model knowledge.^93^ For pesticide monitoring, high uncertainty or out-of-domain inputs should trigger confirmatory measurement, recalibration, or laboratory analysis rather than an unqualified automated decision. Claims of model transferability should consequently be reserved for models that retain acceptable discrimination, concentration error, and uncertainty calibration when evaluated using independent sensors, water matrices, locations, or time periods. Fig. 3 integrates these requirements into a physics-aware analytical workflow linking multimodal sensing, preprocessing, model inference, external validation, uncertainty assessment, and pesticide early-warning systems.

**Fig. 3:**
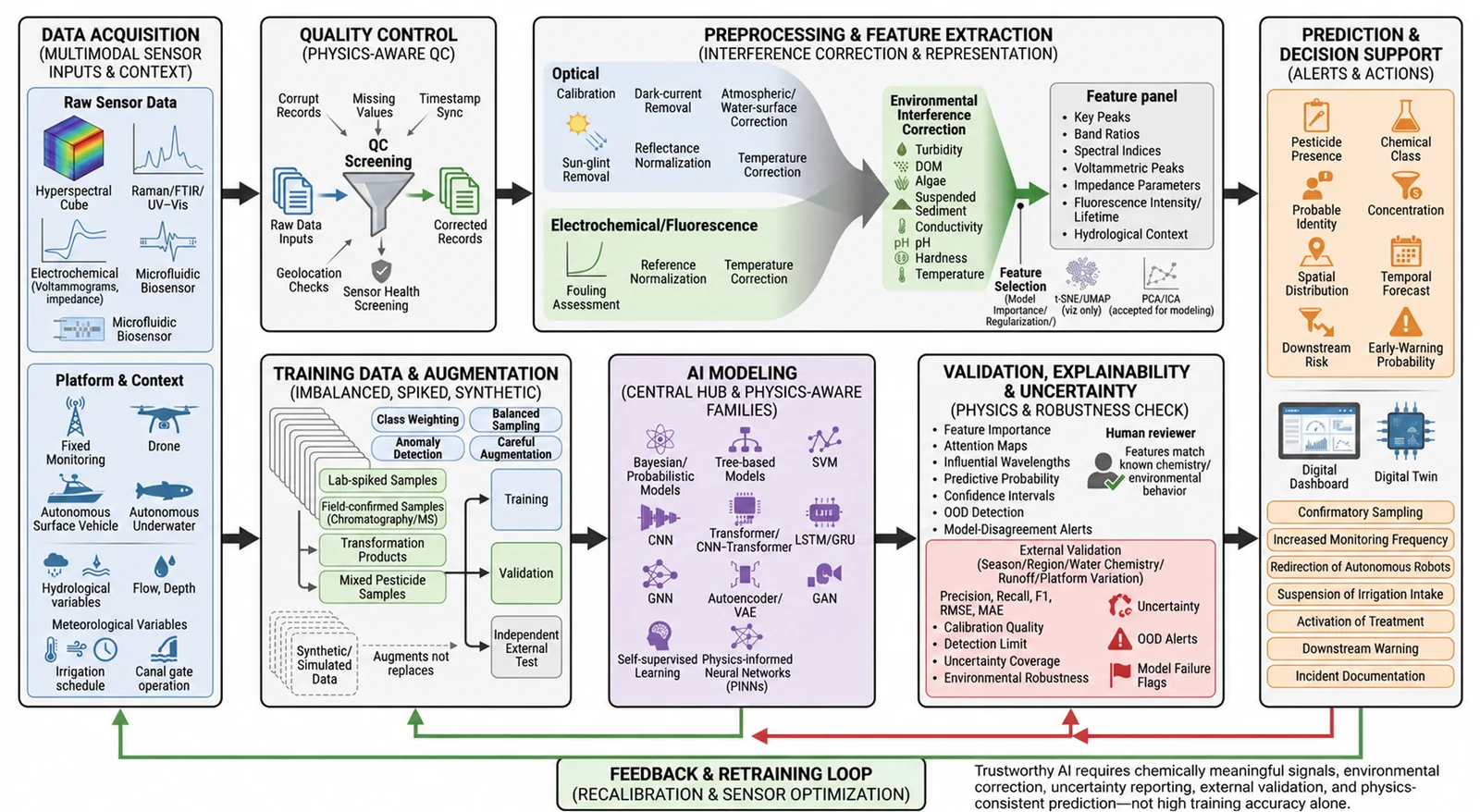
A physics-aware AI pipeline for transforming multimodal water-sensor data into validated pesticide detection, forecasting, uncertainty assessment, and early warning systems. https://app.biorender.com/illustrations/6a73dc9b1a810b4e26140b5d.

## 7 Autonomous aquatic monitoring systems

### 7.1 Autonomous surface and underwater vehicles for intelligent irrigation water monitoring

Self-supervised learning can exploit large quantities of unlabeled or sparsely labeled hyperspectral data by learning useful representations through pretext tasks such as contrastive learning and masked image modeling. These approaches reduce dependence on manually labeled datasets while allowing models to learn complementary spectral–spatial features that can subsequently be fine-tuned for downstream classification tasks. Recent hyperspectral self-supervised frameworks have demonstrated improved representation learning and generalization across multiple datasets, highlighting their potential for data-limited environmental sensing applications.^94,95^ Physics-informed neural networks (PINNs) can become part of data-free autonomous vehicles by incorporating physical laws (*e.g.*, advection, diffusion, and degradation) into the learning process. By limiting the prediction possibilities through the dynamics of hydrology and chemistry, PINNs create physically consistent predictions of pesticide transport and fate, even with minimal or limited data availability and instrumentation. Their capability to integrate sensor data and process-based models makes them particularly useful for the predictive mapping and digital-twin development of irrigation networks for reliable system-level pesticide risk assessment.

### 7.2 Linking pesticide sensors with IoT and autonomous monitoring platforms

IoT and autonomous vehicles function better as transport, communication, and sampling layers for chemical sensors rather than acting as the pesticide detectors themselves. A pesticide-monitoring node must therefore begin with an analyte-specific measurement layer, such as an electrochemical, fluorescence, SERS, or biosensor platform, together with auxiliary measurements that describe the water matrix. For example, the screen-printed glyphosate sensor discussed in Table 1 retained a LOD of 0.03 ppb in surface water, demonstrating a chemically specific sensing element that could potentially be incorporated into a networked monitoring node.^32^ In contrast, pH, conductivity, temperature, and turbidity probes provide contextual variables needed to interpret matrix-dependent changes but do not identify pesticide molecules themselves.^96^ Thus, an IoT architecture becomes relevant to pesticide surveillance only when it acquires, timestamps, georeferences, and transmits pesticide-specific sensor outputs together with the environmental variables required for calibration and quality control. Field-tested water-monitoring systems demonstrate the feasibility of this surrounding architecture, although not yet its routine pesticide-specific implementation. Ref. 97 deployed an IoT platform with multiple mobile sensor nodes for spatially distributed surface-water measurements, integrating sensing, data processing, wireless communications, and autonomous survey planning. Ref. 98 subsequently combined a USV with water-quality sensors and LoRa communication; field tests demonstrated autonomous navigation and wireless monitoring over a working range of approximately 1.5 km. These studies establish that mobile sensing, autonomous sampling, and low-power communications are operationally feasible for water-quality surveillance, but the monitored parameters were conventional water-quality variables rather than pesticide-specific chemical signals.^97,98^ Accordingly, transferring such platforms to pesticide monitoring requires integration with a validated pesticide sensor rather than simply adding AI to an existing robotic vehicle.

A practical pesticide-monitoring workflow would therefore use fixed IoT nodes for high-frequency surveillance at hydrologically important locations, such as drainage outlets or canal junctions, and deploy mobile platforms only when additional spatial resolution is required. Fixed nodes can repeatedly measure pesticide-specific and matrix variables at the same location, whereas an autonomous surface vehicle can revisit a suspected hotspot, map a concentration gradient, or collect confirmatory samples for laboratory analysis.^97,98^ Such integration also requires synchronized timestamps, geolocation, common calibration procedures, standardized units, reference-quality controls, and the explicit handling of missing or drifted sensor values before measurements from fixed and mobile platforms are fused.^67^Fig. 4 summarizes how fixed sensing nodes and aerial, surface, and underwater platforms can be integrated into a pesticide-surveillance architecture while retaining chemical sensors as the primary analytical layer.

**Fig. 4:**
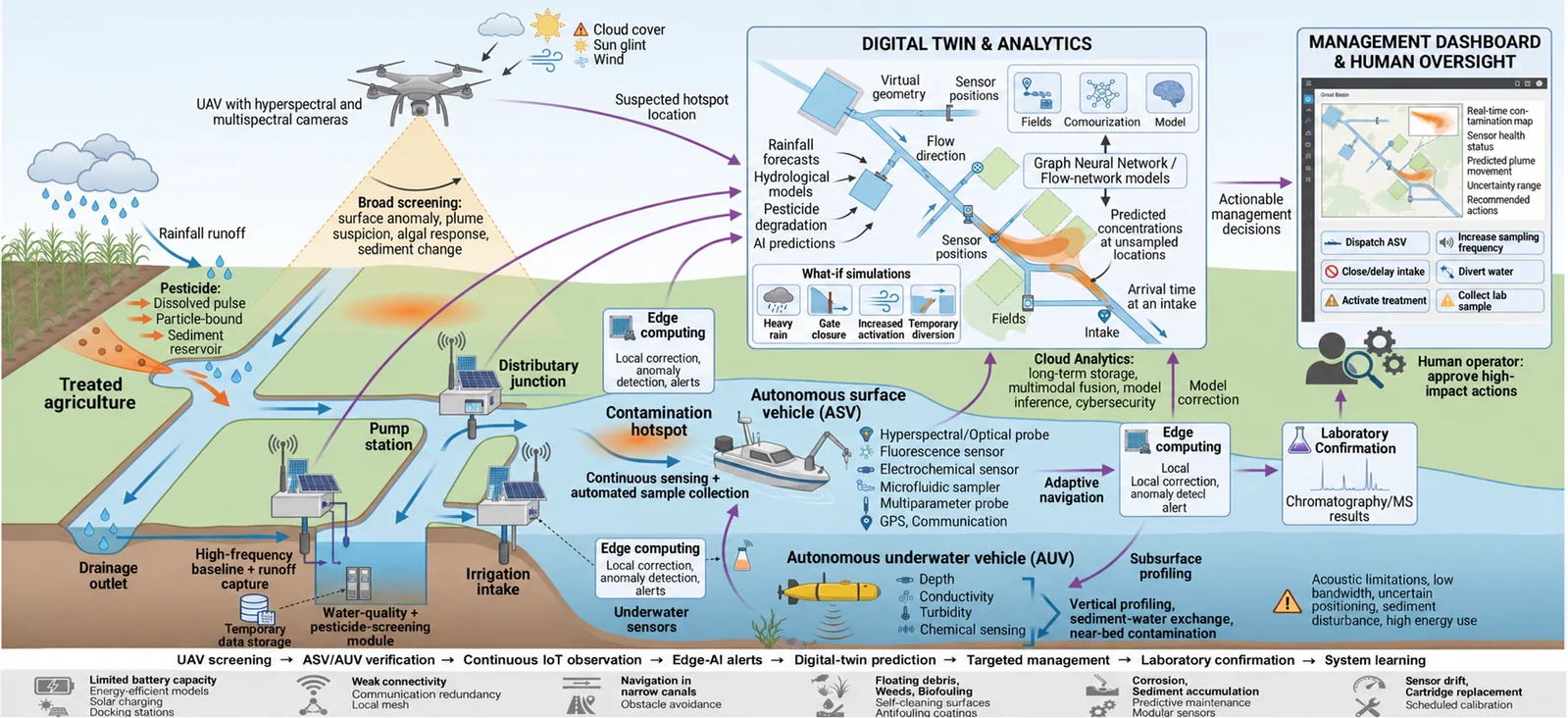
An autonomous aerial, surface, underwater, and fixed-sensor ecosystem for real-time pesticide surveillance and the digital-twin management of irrigation networks. https://app.biorender.com/illustrations/6a73e3afd0fd9298e3c3f7f0.

### 7.3 Digital twins and edge-AI: operational evidence and pesticide-specific readiness

Digital twins are already being implemented in water infrastructure, but pesticide-specific digital twins for agricultural-water surveillance remain at an earlier stage of maturity. Kim *et al.* developed and field-implemented a digital twin for a water-distribution system in Alaska by combining a wireless sensor network with a dynamic hydraulic model to continuously reconstruct pressure, flow, and tank-level states.^99^ This demonstrates the operational feasibility of continuously updated water-system digital twins, but the monitored variables were hydraulic rather than pesticide-specific chemical measurements. Reviews of the broader water sector likewise show growing digital-twin adoption for monitoring and operational decision support, while identifying data integration, model updating, and scalability as continuing challenges.^100^ Consequently, a digital twin that assimilates pesticide-sensor signals, transformation kinetics, hydrological transport, and laboratory confirmation in agricultural irrigation networks should currently be regarded as a prospective pesticide-monitoring architecture rather than an established field technology.

Edge-AI provides an enabling computational architecture, although evidence of pesticide-specific deployment remains limited. Local inference can reduce communication requirements and latency in distributed sensing networks, but existing edge-AI literature is predominantly based on broader smart-city, IoT, and environmental applications rather than validated autonomous pesticide surveillance.^101^ Pruning, low-bit quantization, and task-adaptive hierarchical representations can further reduce memory and communication demands during resource-constrained inference.^102,103^ For pesticide monitoring, edge implementation should therefore be considered operationally plausible but experimentally immature until pesticide-specific models are validated on deployed sensor nodes under realistic water-matrix and power constraints.

For pesticide surveillance, the functions of edge computing and digital twins should therefore remain chemically anchored. Edge inference could screen incoming pesticide-sensor signals for anomalous responses, apply previously validated matrix corrections, and determine whether data should trigger higher-frequency sampling or laboratory confirmation; however, such decisions remain credible only when the underlying pesticide sensor has been independently validated in the target water matrix.^9,32^ At the network level, a future pesticide digital twin would assimilate these verified concentration measurements with flow, rainfall, application timing, and pesticide-specific degradation or transport parameters, rather than relying only on generic water-quality variables. The operational feasibility of continuously updated hydraulic digital twins has been demonstrated in water-distribution systems, but pesticide-specific assimilation remains prospective.^99^

### 7.4 Practical requirements for pesticide-specific autonomous deployment

The principal deployment criterion is the analytical validity of a sensor during its deployment in a mobile or networked system. The potential to co-design perception and movement in autonomous platforms is also demonstrated in integrated actuation-sensing architectures in soft robotics.^104^ Controlled flow, contact time, sample conditioning, reagent delivery, electrode renewal and periodic calibration are some of the requirements of pesticide-specific sensors, which may not suit the low-power and unattended operation that is expected from IoT nodes and autonomous vehicles.^41,96^ A number of other factors such as biofouling, the deposition of sediment and the ageing of sensors, also necessitate cleaning or recalibration strategies – transmitting a drifted signal more quickly does not improve monitoring quality .^67^ There is also a need to synchronise communication and energy needs with the analytical task. The feasibility of distributed aquatic telemetry has been revealed by the use of LoRa-based USV monitoring, which has shown low-power, kilometre-scale transmission for traditional water-quality sensing.^98^ However, duty cycling or event-triggered operation may be more desirable than continuous full-power measurement of pesticides when employing excitation sources, pumps, microfluidics, spectral acquisition, or onboard AI, as this could result in significantly higher energy requirements.^98,105^

Recent analysis of solar-powered IoT water-monitoring systems likewise shows that most field deployments still concentrate on pH, turbidity, conductivity, temperature, and dissolved oxygen, whereas the autonomous monitoring of specific chemical contaminants remains substantially less mature.^105^ Finally, a system should define what happens after a pesticide alert. A defensible workflow begins with sensor measurement and matrix-specific quality-control checks, followed by edge- or cloud-based inference and uncertainty assessment. Where uncertainty remains high, or when an alert is generated, the system should initiate repeat measurement or targeted mobile sampling, with confirmatory chromatographic analysis performed when required.^9,15^

## 8 External validation, benchmarking, and deployment performance

### 8.1 Validation hierarchy and performance metrics for pesticide-sensing AI

Benchmarking should distinguish internal model fitting from evidence of generalization. Calculating performance based on training data provides no independent assessment, while random hold-out testing and conventional *k*-fold cross-validation provide only internal validation when samples originate from the same experimental population. For environmentally structured data, grouped validation that withholds complete locations, sampling periods, sensor units, or experimental batches provides a stronger test because correlated observations are prevented from appearing in both training and testing sets.^71,72^ The strongest evidence for deployment is independent external validation using samples collected outside the model-development dataset and, ideally, under different water-matrix, sensor, spatial, or temporal conditions. Classification accuracy should not be used as the principal measure when contamination events are uncommon. A model can achieve high overall accuracy by correctly identifying abundant uncontaminated samples while failing to detect the minority pesticide class. Precision, therefore, quantifies the reliability of positive alerts, whereas recall measures the proportion of actual contamination events detected; *F*1 integrates both quantities, and the Matthews correlation coefficient provides a balanced measure when class frequencies differ strongly.^106,107^ Confusion matrices and class-specific sensitivity should consequently accompany overall accuracy whenever AI is proposed for early-warning surveillance.

Concentration-estimation models require complementary regression metrics. MAE describes the average absolute prediction error, whereas RMSE gives greater influence to larger errors and may therefore expose occasional severe concentration-estimation failures.^108^*R*^2^ describes explained variance but should not be interpreted as a direct measure of prediction error, and concentration predictions should therefore be accompanied by MAE or RMSE and an examination of systematic bias. Where uncertainty estimates are produced, their calibration and coverage based on independent data should also be evaluated because accurate point estimates alone do not establish that a model recognizes unreliable or out-of-domain predictions.^93^

### 8.2 Domain shift, sensor drift, and deployment robustness

Robustness testing should reproduce the distribution shifts expected during deployment rather than adding arbitrary synthetic noise alone. Pesticide models intended for agricultural water should therefore be challenged across relevant ranges of turbidity, DOM, pH, ionic strength, temperature, suspended solids, degradation products, and co-contaminants because these factors can alter both the chemical form of the analyte and the sensor signal.^9,19^ Performance should be reported separately for in-domain samples and deliberately withheld matrix conditions so that degradation under environmental domain shifts is visible rather than averaged into a single accuracy value.

Transfer across sensor units and deployment time represents an additional validation level. Chemical sensors can undergo baseline and sensitivity drift, while environmental exposure may produce fouling-related changes that were absent during laboratory calibration.^67,92^ A model described as transferable should therefore be evaluated first on a different sensor unit or sensor batch and again after an appropriate deployment interval. Domain adaptation or recalibration should be evaluated by comparing performance before and after adaptation on an independent target-domain test set, rather than reporting improvement on the samples used to update the model.^91^ Deployment benchmarking should finally combine analytical performance with computational feasibility. Inference time, memory demand, energy consumption, communication requirements, and missing-data resilience are relevant for edge devices and autonomous platforms, but computational efficiency has practical value only if acceptable pesticide-detection performance is retained under external validation.^15,109^ Thus, a lightweight model with independently validated performance may be more useful for continuous surveillance than a larger model that achieves marginally higher accuracy only on an internal laboratory test set.

Laboratory analytical sensitivity and field readiness should therefore be reported as separate performance dimensions. LODs obtained from standards in buffer solution or ultrapure water establish analytical proof-of-concept but do not demonstrate equivalent performance in environmental water, where matrix interference, fouling, drift, and variable physicochemical conditions may modify both sensitivity and selectivity.^67,110^ Real-matrix validation should report recovery, bias, precision, and agreement with a reference analytical method, whereas true field validation additionally requires the assessment of signal stability, false-alert frequency, sensor drift, maintenance requirements, and performance across changing hydrological and water-quality conditions.^96,110^ Accordingly, laboratory LODs, real-sample analytical performance, and sustained *in situ* performance should not be presented as interchangeable evidence of monitoring capabilities. Table 2 summarizes this validation hierarchy together with classification, regression, uncertainty, robustness, and deployment benchmarks for pesticide-sensing AI.

**Table 2:** Validation hierarchy, predictive metrics, robustness criteria, and deployment benchmarks for AI-assisted pesticide sensing

S. no.	Metric/benchmark	Category	What it measures	Application/relevance in pesticide detection	Reference
1	Overall accuracy (OA)	Classification	Percentage of correctly classified samples	General indicator of classifier performance toward spectral data	111
2	Average accuracy (AA)	Classification	Mean accuracy across all classes	Important for imbalanced datasets with rare contamination events	112
3	Kappa coefficient (*κ*)	Classification	Agreement beyond random chance	More reliable metric when class frequencies are unequal	113
4	Precision	Classification	Correct positive predictions out of predicted positives	Critical for avoiding false contamination alerts	107
5	Recall (sensitivity)	Classification	Correct positive predictions out of actual positives	Important for detecting low-concentration pesticide events	107
6	*F*1 score	Classification	Harmonic mean of precision and recall	Balanced indicator for rare pesticide contamination detection	106
7	Spectral angle mapper (SAM)	Spectral similarity	Angle between spectral vectors	Robust against illumination variability in water bodies	114
8	Spectral information divergence (SID)	Spectral similarity	Divergence between probability distributions of spectra	Helps differentiate chemically similar pesticides	115
9	Root mean square error (RMSE)	Regression	Magnitude of prediction errors	Used in concentration estimation models for pesticides	108 and 116
10	Mean absolute error (MAE)	Regression	Mean absolute deviation between predicted and true values	Less sensitive to outliers in concentration prediction	108
11	Coefficient of determination (*R*^2^)	Regression	Variance explained by the regression model	Indicates model fit for concentration estimation	117
12	Mean absolute percentage error (MAPE)	Regression	Percentage deviation between observed and predicted values	Useful when pesticide levels span wide concentration ranges	118
13	Blocked/grouped cross-validation	Internal validation	Generalization when complete locations, times, batches, or sensor groups are withheld	Reduces information leakage from correlated pesticide-sensor observations	71 and 72
14	Independent external validation	External validation	Performance on data collected independently of model development	Strongest evidence for transfer to new water matrices, locations, seasons, or sensors	71
15	Domain-shift performance degradation	Robustness	Change in predictive performance between source-domain and independent target-domain data	Quantifies sensitivity to matrix, site, or sensor changes	91
16	Uncertainty calibration/coverage	Uncertainty	Agreement between predicted uncertainty and observed predictive error	Identifies unreliable or out-of-domain pesticide predictions	93
17	Inference time	Hardware performance	Time required for a single prediction	Critical for real-time ASV-/AUV-based monitoring	119
18	Energy consumption per inference	Hardware performance	Power used per prediction	Important for edge-AI deployment on battery-limited platforms	119
19	Memory footprint	Hardware performance	RAM or storage required by the model	Determines feasibility for IoT nodes or embedded processors	109
20	Scalability and throughput	System-level	Ability to handle increasing data volume or sensor count	Required for large-scale irrigation network monitoring	120
21	Real-matrix recovery and reference-method agreement	Analytical validation	Bias, recovery, precision, and agreement when analysing environmental water rather than standards	Determines whether laboratory sensitivity survives realistic matrix interference	110
22	*In situ* stability and operational uptime	Field validation	Signal stability, drift, fouling, maintenance frequency, and usable monitoring time during deployment	Distinguishes short laboratory testing from sustained environmental surveillance	67

## 9 Case studies and real-world deployment

### 9.1 From laboratory sensitivity to real-matrix and field performance

Evidence of pesticide-sensor performance can be separated into four levels: controlled analytical standards, spiked environmental matrices, authentic environmental samples analysed *ex situ*, and sustained *in situ* field deployment. These levels provide progressively stronger evidence because performance obtained from purified standards does not incorporate the chemical interference, fouling, environmental variability, and operational instability encountered during real monitoring.^96,110^ Consequently, the terms “real-sample validation,” “on-site measurement,” and “field deployment” should not be used interchangeably: analysis of an environmental sample in the laboratory demonstrates matrix applicability, whereas field deployment requires the sensor to operate reliably under changing environmental conditions. The difference is evident among the representative pesticide sensors summarized in Table 1. The SERS detection of thiram showed a LOD of 1.0 × 10^−11^ M in aqueous solution, demonstrating exceptional analytical sensitivity but not performance in complex agricultural water.^33^ A molecularly imprinted fluorescent sensor for imidacloprid achieved a LOD of 0.3 nM and a response within 1 min and was tested in spiked environmental water, providing stronger evidence of matrix compatibility but still not continuous *in situ* performance.^30^ By comparison, a glyphosate electrochemical platform retained a LOD of 0.03 ppb in surface water and produced recoveries of 93.8–98.9%, demonstrating that analytical sensitivity could be maintained in a realistic water matrix under *ex situ* testing.^32^

Authentic natural-water studies also reveal limitations that may not be apparent from laboratory LODs. Reflectometric atrazine sensing achieved a LOD of 0.35 µg L^−1^ and a 15 min analysis time in natural-water application, but humic substances caused substantial interference and structurally related triazines produced cross-reactivity.^25^ Multivariate fluorescence analysis similarly demonstrated that imidacloprid could be quantified in real water samples containing fluorescent pesticides and unexpected interferents, illustrating the value of matrix-aware calibration rather than relying on sensitivity alone.^26^ Importantly, the representative pesticide studies summarized in Table 1 predominantly demonstrate laboratory, spiked-matrix, or *ex situ* real-water performance rather than unattended long-term deployment in irrigation networks. This gap remains central to the translation of pesticide sensors from analytical prototypes to operational surveillance systems, where drift, fouling, calibration stability, maintenance, and agreement with reference methods become as important as nominal LODs.^67,110^ System-scale readiness therefore requires evidence not only that a pesticide sensor functions in real water, but also that its calibration, sampling protocol, telemetry, power supply, quality-control procedures, and confirmatory workflow remain reliable when the sensor is incorporated into fixed or mobile monitoring infrastructure.^96,105^

### 9.2 Commercial *versus* prototype sensing systems and economic viability in irrigation monitoring

Commercial aquatic sensors are now mature and reliable, and simple to deploy. However, they are largely restricted to general water-quality measurements such as pH, turbidity, conductivity and dissolved oxygen. These sensors do not directly detect pesticides. In contrast, sensing systems under development in the prototype stage include fluorescence, surface-enhanced Raman scattering, microfluidics, and nanomaterial-enhanced electrochemical platforms that can be used to detect trace pesticide concentrations with high sensitivity and specificity.^96^ These prototypes face a number of challenges. They often have problems with long-term stability, calibration, environmental interference and large-scale manufacturability. Field comparisons, typically using autonomous surface vehicles or undersea vehicles, highlight trade-offs between various parameters related to performance, robustness, maintenance and scale-up. These comparisons aid in coming up with pathways toward commercialization.^121^ Economic feasibility is an important factor that determines adoption in the real world. Cost-benefit analyses are required to take sensor hardware, operation, installation and maintenance, power provision, data communication, and platform running into account, particularly for mobile robotic systems. Even though initial costs may be high, they are typically compensated for by the benefits of the early detection of pesticides. These benefits include less crop loss, lower water-treatment costs, regulatory compliance and the prevention of long-term environmental damage. Advances in low-cost electronics and open-source and energy-efficient designs are further improving the aspects of scaling and affordability. As a result, integrated monitoring systems are becoming more attractive for use in large and/or high-value irrigation schemes^122^ and monitoring systems of natural resources.

## 10 Challenges and research gaps

A major challenge in AI-based pesticide detection is the limited availability of high-quality labeled data covering all irrigation-water conditions. Obtaining appropriate ground-truth data is expensive, and it requires laboratory analysis and well-regulated spiking experiments. To make matters worse, environmental effects like turbidity, algae, dissolved organic matter and patchy sediment may alter the spectral signatures, making labeling more difficult. The limited availability of labeled data makes it difficult for models to generalize and increases their risk of overfitting, particularly when detecting rare or novel pesticides.^123^ The chemical structures of many pesticides are similar, leading to overlapping spectral features that even advanced deep learning models cannot resolve. The minor spectral variations between neonicotinoids, organophosphates and phenylureas have been found to be lost under noisy or convoluted water conditions. In order to make a distinction between structurally related pesticides, we require the most sensitive sensors, preprocessing pipelines that can improve features, and models that can capture an observable fine-grained chemical signature.^83^ Turbidity, algal blooms, temperature, depth, and dissolved organic matter influence irrigation water, coloring its spectral and electrochemical signals in a distorted manner. Those models that are trained exclusively in controlled or limited circumstances may not be reliable for use in different seasons, regions, or hydrological events. We require good environmental normalization techniques and domain-adaptation techniques to obtain reliable performance in a wide variety of real-world environments.^124^ Both hyperspectral and multi-sensor data have very high dimensionality. This dimensionality escalation increases the computational cost and the probability of overfitting. Without the proper regularization of deep learning models, noise can be learned in addition to significant chemical patterns. We should have feature-reduction algorithms, self-supervised pretraining, and lightweight models in order to shrink the dimensionality without decreasing the accuracy.^125^

Pesticide detection with AI, in contrast to conventional water-quality monitoring, has no established evaluation procedures so far. Differences in sensor hardware, sensor calibration and environmental conditions make it challenging to compare outputs across studies. Another urgent requirement is a global standard that covers a broad range of pesticides, concentrations, and environmental conditions, to establish plausible validation systems and accelerate regulatory acceptance.^126^ Infrastructure issues like low power reliability, lack of connectivity, excessive sediment loads and limited technical capacity to maintain irrigation systems are challenges faced by irrigation networks in various developing regions. Autonomous robots and advanced sensors tend to wear out easily in harsh environments. One of the research gaps that has to be filled is the development of low-cost, robust, and easily maintainable systems to achieve even distributions of adoption and sustainability.^126^

## 11 Future research directions

Next-generation quantum and plasmonic sensors can detect pesticides at the parts-per-trillion level, and these are more sensitive than any other method. To discover vibrational signatures, they employ quantum dots, plasmon-enhanced Raman substrates, and nanoscale waveguides well below the time constraints of classical spectral spectroscopy. These sensors should be refined for long-term use in aquatic environments and combined with autonomous platforms for continuous and real-time monitoring.^127^ Large-scale foundation models that are trained on huge libraries of spectral, chemical, and environmental data can be extrapolated across regions and sensors, as well as across classes of pesticides. Similar to language or visual foundation models, spectral transformer and multi-modal AI models may provide universal embeddings for water-quality analysis. The construction of these models will need worldwide data-sharing programs and programmed regularizations of the preprocessing pipelines.^128^ The use of AI in water monitoring depends on transparent and interpretable predictions. Explainable AI may reveal what wavelength ranges, chemical features or environmental variables are used to make decisions concerning contamination. Regulatory approval, user trust, and the improvement of sensing strategies are facilitated by constructing interpretable models by identifying the spectral features of greatest impact.^129^

We can predict contamination events by using hyperspectral images, *in situ* chemical sensors, autonomous vehicle measurements, hydrological modeling, and weather predictions in one spatiotemporal model. Multimodal models of this kind are used to predict pesticide spikes caused by runoff, to monitor the movement of plumes and to create more efficient methods to schedule sensor deployment.^130^ Federated learning allows institutions or regions to train models without exchanging raw data. This approach is particularly appropriate in pesticide monitoring cases, where privacy, regulatory requirements, and the variability of the data limit the sharing of the data. Federated systems have the potential to produce globally robust models that remain environmentally adaptive while preserving local data ownership.^131^ Future sensing platforms should be low-cost, sediment-resistant, biofouling-resistant, and low-power to ensure their widespread use. The use of printable electrochemical sensors, biodegradable microfluidics, solar-powered ASVs, and low-cost optical modules can help revolutionize irrigation monitoring in resource-constrained areas. The materials most desirable in research include those that are resistant to biofouling, those that use self-cleaning surfaces, and those that provide modular sensor components that can be implemented on a large-scale basis.^132^ Real-time decision-making artificial intelligence will be incorporated into future ASVs and AUVs. They will take sampling paths depending on the contamination gradient, hydrological conditions or AI forecasts. These robots would be able to work in swarms, and they would evenly distribute the work. This strategy is most effective in terms of maximum spatial coverage and the reduction of redundancy. Adaptive navigation strategies that follow contamination likelihood maps will be made possible through reinforcement learning.^133^ Digital twins of irrigation systems will turn into high-fidelity platforms involving hydrodynamic models, pesticide degradation models, robotic navigation models, and AI-based inference. They will be able to run what-if scenarios, enhance pesticide-detection programs, and simulate the impact of climate change on the movement of chemicals. Cross-domain dual-engine AI frameworks also demonstrate the potential of coupling predictive models with iterative optimization.^134^ They will no longer be used to monitor operations but to support strategic planning, irrigation scheduling and regulatory work.^135^ Spatial-scale uncertainty and multicriteria evaluation can further support the translation of environmental information into risk-based management decisions.^136,137^ The resulting priorities for analytical validation, external benchmarking, field translation, system integration, and regulatory implementation are collected in Fig. 5.

**Fig. 5:**
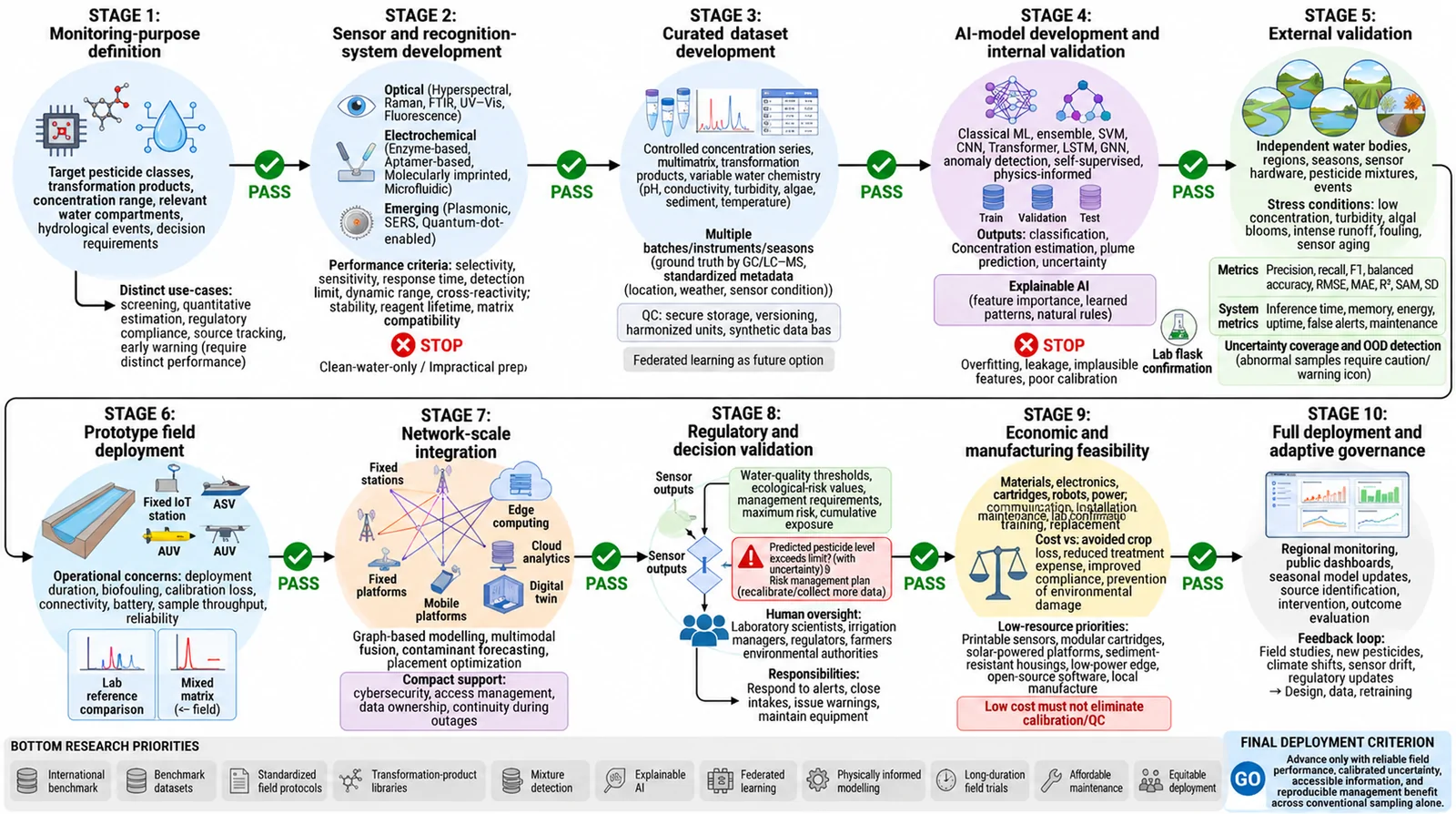
An evidence-based roadmap for validating, benchmarking, scaling, regulating, and deploying AI-enabled pesticide-monitoring systems in agricultural water networks. https://app.biorender.com/illustrations/6a73e52208d7fd36bbe6bd05.

## 12 Conclusions

The integration of pesticide-specific sensing, artificial intelligence, and autonomous monitoring provides a promising framework for improving the surveillance of agricultural water systems. Optical spectroscopy, fluorescence, electrochemical sensors, biosensors, microfluidic platforms, and multimodal sensing provide complementary analytical information, although their practical value depends on the pesticide chemistry, matrix tolerance, calibration stability, and real-water validation. AI can support classification, concentration estimation, matrix correction, multimodal fusion, anomaly detection, and forecasting when the underlying sensor information is sufficiently informative and when models are validated against appropriate conventional baselines. However, advanced architectures should not be assumed to provide superior performance solely because of their computational complexity. Their value depends on independent validation, robustness to environmental domain shifts, uncertainty assessment, and transferability across sensors and water matrices. Current evidence also reveals a substantial gap between laboratory sensitivity and operational deployment. Many pesticide-sensing studies remain limited to controlled standards, spiked matrices, or *ex situ* analysis, whereas sustained autonomous monitoring requires the management of sensor drift and biofouling, as well as reliable power supply and communications, stable calibration, and confirmatory analytical procedures. IoT networks, autonomous vehicles, edge computing, and digital twins can extend the spatial and temporal coverage of pesticide surveillance, but these technologies should be regarded as supporting infrastructure around validated chemical sensors rather than as pesticide-detection technologies themselves. Future progress therefore depends on chemistry-aware sensing, representative training datasets, independent field validation, standardized benchmarking, and the transparent integration of sensor measurements with AI-based decision support.

## Author contributions

H. K. and M. A. conceived the review concept and designed the overall framework. H. K. conducted the literature survey, synthesized the scientific evidence, prepared the original manuscript draft, and secured funding for the study. S. I. contributed to the artificial intelligence, machine learning, and software-related sections and critically reviewed the manuscript. A. H. A. contributed regarding environmental monitoring systems, engineering perspectives, and manuscript revision. N. U. S. assisted in data interpretation, visualization, and the preparation of figures and tables. R. N. contributed regarding computational methodologies, technical discussions, and critical editing of the manuscript. J. I. provided intellectual input, supervised the environmental sciences perspective, and critically revised the manuscript. W. A. K and K. H. K. contributed to literature analysis, visualization, proofreading, and manuscript editing. M. A. supervised the study, coordinated manuscript preparation, and critically revised the final version. All authors read, revised, and approved the final manuscript. Writing, review & editing: all authors contributed to manuscript improvement.

## Conflicts of interest

The authors declare no conflicts of interest.

## Funding

This work was supported by the Deanship of Scientific Research, Vice Presidency for Graduate Studies and Scientific Research, King Faisal University, Saudi Arabia [Grant No. KFU266208].

## Data Availability

No new experimental data were generated or analysed in this review. All information discussed in the manuscript was obtained from the previously published literature cited within the article.
